# Host-derived protease promotes aggregation of *Staphylococcus aureus* by cleaving the surface protein SasG

**DOI:** 10.1128/mbio.03483-23

**Published:** 2024-03-21

**Authors:** Heidi A. Crosby, Klara Keim, Jakub M. Kwiecinski, Christophe J. Langouët-Astrié, Kaori Oshima, Wells B. LaRivière, Eric P. Schmidt, Alexander R. Horswill

**Affiliations:** 1 Department of Immunology and Microbiology, University of Colorado School of Medicine, Aurora, Colorado, USA; 2 Department of Microbiology, Faculty of Biochemistry, Biophysics and Biotechnology, Jagiellonian University, Krakow, Poland; 3 Division of Pulmonary Sciences and Critical Care Medicine, Department of Medicine, University of Colorado School of Medicine, Aurora, Colorado, USA; 4 Division of Pulmonary Sciences and Critical Care, Department of Medicine, Massachusetts General Hospital, Boston, Massachusetts, USA; 5 Department of Veterans Affairs Eastern Colorado Health Care System, Denver, Colorado, USA; University of Washington, Seattle, Washington, USA

**Keywords:** *Staphylococcus aureus*, MRSA, SasG, lung infection, aggregation

## Abstract

*Staphylococcus aureus* is one of the leading causes of hospital-acquired infections, many of which begin following attachment and accumulation on indwelling medical devices or diseased tissue. These infections are often linked to the establishment of biofilms, but another often overlooked key characteristic allowing *S. aureus* to establish persistent infection is the formation of planktonic aggregates. Such aggregates are physiologically similar to biofilms and protect pathogens from innate immune clearance and increase antibiotic tolerance. The cell-wall-associated protein SasG has been implicated in biofilm formation via mechanisms of intercellular aggregation but the mechanism in the context of disease is largely unknown. We have previously shown that the expression of cell-wall-anchored proteins involved in biofilm formation is controlled by the ArlRS-MgrA regulatory cascade. In this work, we demonstrate that the ArlRS two-component system controls aggregation, by repressing the expression of *sasG* by activation of the global regulator MgrA. We also demonstrate that SasG must be proteolytically processed by a non-staphylococcal protease to induce aggregation and that strains expressing functional full-length *sasG* aggregate significantly upon proteolysis by a mucosal-derived host protease found in human saliva. We used fractionation and N-terminal sequencing to demonstrate that human trypsin within saliva cleaves within the A domain of SasG to expose the B domain and induce aggregation. Finally, we demonstrated that SasG is involved in virulence during mouse lung infection. Together, our data point to SasG, its processing by host proteases, and SasG-driven aggregation as important elements of *S. aureus* adaptation to the host environment.

**IMPORTANCE:**

Here, we demonstrate that the *Staphylococcus aureus* surface protein SasG is important for cell-cell aggregation in the presence of host proteases. We show that the ArlRS two-component regulatory system controls SasG levels through the cytoplasmic regulator MgrA. We identified human trypsin as the dominant protease triggering SasG-dependent aggregation and demonstrated that SasG is important for *S. aureus* lung infection. The discovery that host proteases can induce *S. aureus* aggregation contributes to our understanding of how this pathogen establishes persistent infections. The observations in this study demonstrate the need to strengthen our knowledge of *S. aureus* surface adhesin function and processing, regulation of adhesin expression, and the mechanisms that promote biofilm formation to develop strategies for preventing chronic infections.

## INTRODUCTION

*Staphylococcus aureus* asymptomatically colonizes the nostrils, throat, and skin of ~30% of the population, and a portion also carries *S. aureus* in their oral cavity (1–5). Nasal carriage is a significant risk factor for developing nosocomial infections (6, 7), with ~80% of infections caused by the patient’s colonizing strain (8–10). *S. aureus* is one of the leading causes of healthcare-associated infections, such as surgical site infections and central line-associated bloodstream infections (11), imposing a substantial burden on the healthcare system. While these infections are often challenging to treat, the rise of methicillin-resistant *S. aureus* (MRSA), which causes over 119,000 of these infections annually in the United States, has further exacerbated treatment challenges and increased healthcare costs by nearly one billion dollars annually (12–15).

*S. aureus* is one of the most prevalent pathogens in chronic wound infections (16–18) and is one of the first pathogens to colonize the cystic fibrosis (CF) lung (19). The occurrence of chronic and persistent *S. aureus* infections is in part due to aggregation mechanisms and the ability of this pathogen to adhere to indwelling medical devices as a biofilm (20, 21). However, in the absence of an implanted medical device, *S. aureus* can form free-floating aggregates that are physiologically similar to biofilms and are likewise more antibiotic resistant (22, 23). It has been suggested that bacterial aggregates predominate in chronic infections such as osteomyelitis (24), chronic wounds (25), and the lungs of CF patients (26, 27). Intensive efforts to clear MRSA lung infections in CF patients, sometimes using up to five different antibiotics, have shown some promise, although ~15% of patients still harbor MRSA at the end of the intervention period (28–30). A better understanding of *S. aureus* biofilm formation and aggregation may lead to alternative therapies for these difficult-to-treat infections.

MRSA aggregation observed in clinical infections has been described as groups of closely attached cells that are not surface attached, and similarly to mature biofilms, they protect from environmental stress and allow for persistence (22). Aggregates and biofilms are difficult to treat in part because they are up to 1,000-fold more resistant to antibiotics than planktonic cells (22, 31, 32). This increased tolerance is thought to be due to a combination of slowed diffusion of antibiotics through the extracellular matrix and slower growth of cells within the community of cells (33). In addition, aggregates are more resistant to clearance by the innate immune system, in part due to their large size, which impedes phagocytosis, and their ability to secrete and concentrate toxins that target leukocytes (34–37).

**Fig 1:**
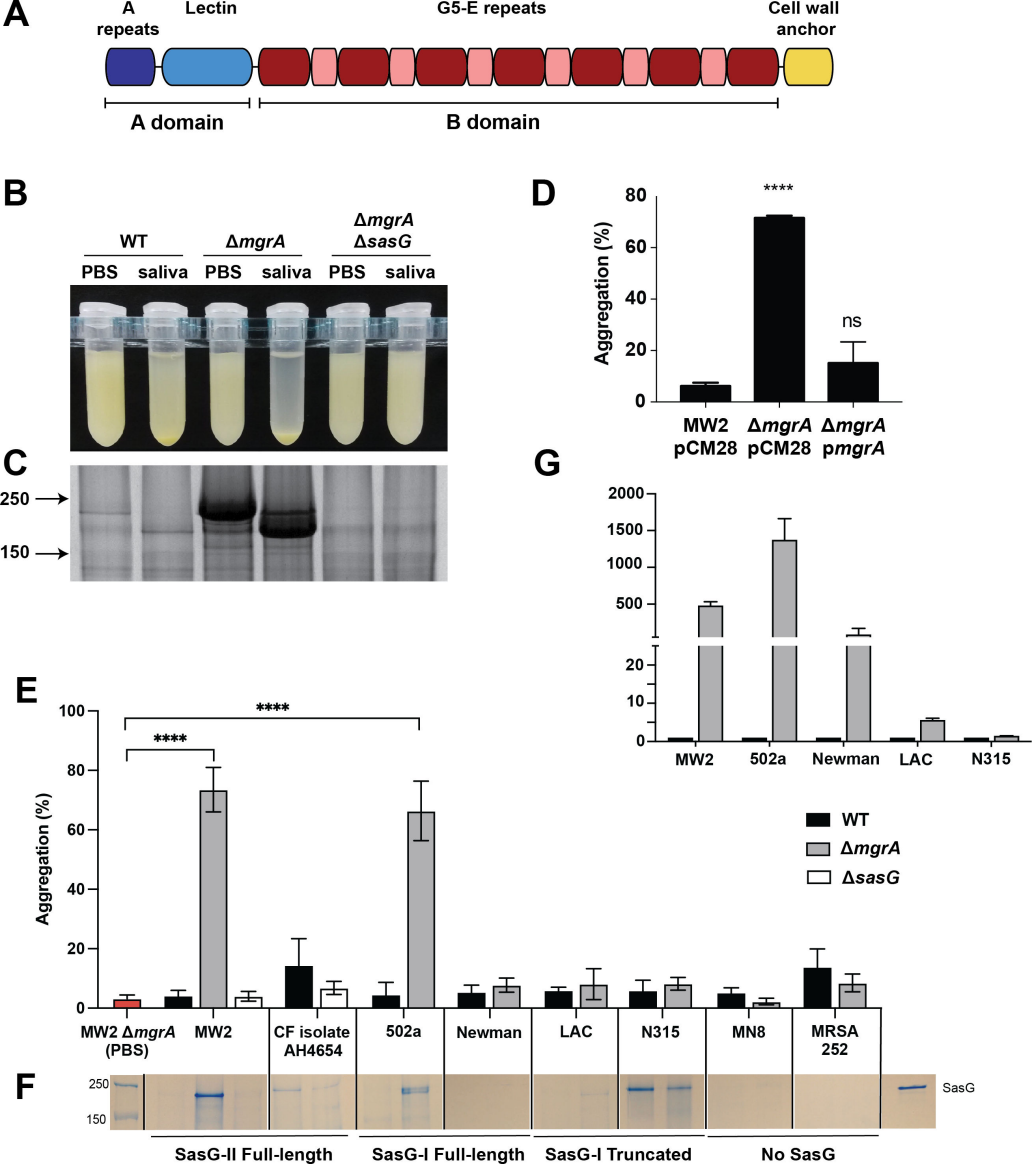
*S. aureus* aggregates in the presence of human saliva and high SasG levels. (**A**) Schematic of SasG domains. (**B–D**) Overnight cultures of the indicated MRSA MW2 strains were spun down and resuspended in either phosphate-buffered saline or clarified human saliva. (**B**) The photo shows an aggregation of the Δ*mgrA* mutant after 1 hour of incubation at room temperature. (**C**) Coomassie-stained SDS-PAGE gel shows cell wall preps from these same samples after 1-h incubation as described above. The experiment is representative of at least three replicates. (**D**) Quantification of aggregation of MW2 WT with the empty vector pCM28, or Δ*mgrA* mutant with either pCM28 or the complementation vector pCM28-*mgrA* (pHC66) in the presence of saliva. Data represent averages and standard deviations of three separate experiments. Statistical significance was calculated by One-way ANOVA. ****, *P* ≤ 0.0001; ns, not significant. (**E**) Various *S. aureus* strains with full-length, truncated, or lacking *sasG* were incubated with human saliva and aggregation was measured following 2 h of incubation. (**F**) Cell wall proteins were precipitated from overnight cultures and run on SDS PAGE to observe relative SasG expression levels. (**G**) Quantification of *sasG* gene expression of various *S. aureus mgrA* mutant strains relative to the respective wild-type *sasG* expression (*n* = 3). Values are normalized to *gyrB* expression in each strain.

One of the key drivers of biofilm formation and aggregation in *S. aureus* is the large, cell-wall-attached surface protein G (SasG) (38–40). SasG and its *S. epidermidis* homolog Aap consist of multiple domains with distinct functions (Fig. 1A). The A domain, which has 59% identity to Aap, is implicated in binding to corneocytes (41) and nasal epithelial cells (42), and has a short, variable repeat region and an L-type lectin subdomain. In full-length SasG, the B domain, which has 60%–67% identity to Aap depending on B-repeat number, consists of 2–17 repeats of alternating G5 subdomains and E spacers (38, 43, 44). These G5-E repeats can dimerize in a Zn-dependent manner to form a twisted cable structure that facilitates intercellular interactions (45). In *S. epidermidis*, the Aap A domain is removed by the metalloprotease SepA, allowing the exposed B domains to dimerize and promote biofilm accumulation (46). Exogenous addition of the host proteases trypsin and cathepsin G can also enhance *S. epidermidis* biofilm formation through the processing of Aap (43). Whether SasG also needs to be proteolytically processed is not known, although it appears that none of the known proteases secreted by *S. aureus* can specifically target SasG (38).

Expression of *sasG* is variable across *S. aureus* clinical isolates. SasG is constitutively expressed by some clinical isolates (39), and the presence of anti-SasG human antibodies demonstrates its expression during infection (47, 48). While the majority of *S. aureus* strains encode *sasG* (49), some do not express it under laboratory conditions (39, 50). Recently, it has become apparent that this lack of SasG expression might be due to its repression by an ArlRS-MgrA regulatory cascade under *in vitro* conditions (48, 51).

In this project, we took advantage of the high level of SasG expression in certain *S. aureus* strains to investigate the role of SasG in aggregation and virulence. We identified that the presence of SasG increases *S. aureus* virulence during lung infection and that the cleavage of the N-terminal portion of the A domain of SasG is necessary for *S. aureus* to aggregate. Since *S. aureus* does not cleave SasG on its own (38), SasG cleavage during infection must be mediated by host proteases. Such cleavage leads to SasG-mediated aggregation of *S. aureus*, which is reflected in increased virulence of SasG-expressing strain during lung infection. Overall, the host-driven cleavage of SasG establishes an unusual and novel way of sensing and responding to the host environment.

## RESULTS

### SasG saliva interaction and expression levels across *S. aureus* strains

Aspiration of saliva is often a precursor to lung infections (52–55), leading us to investigate how MRSA reacts to the presence of human saliva. We made a somewhat surprising observation that a USA400 MRSA Δ*mgrA* mutant strain aggregated to high levels when the cells were resuspended in human saliva, while the WT strain remained in suspension (Fig. 1B). Knowing there is differential surface protein expression in Δ*mgrA* mutants (48), we ran Coomassie protein gels (Fig. 1C) and observed dramatic salivary processing of a large protein that we reasoned might be surface protein G (SasG). Upon constructing a MRSA Δ*mgrA* Δ*sasG* double-mutant, the protein and aggregation phenotype both disappeared (Fig. 1B and C), demonstrating this phenotype is due to SasG. In addition, the aggregation could be complemented by providing *mgrA* on a plasmid (Fig. 1D).

We next investigated the generality of this phenotype in *S. aureus*. We compared sequenced *S. aureus* strains containing functional chromosomal copies of *sasG* including community-acquired MRSA (CA-MRSA) USA400 strain MW2, Newman, 502 a, and a CF clinical MSSA isolate AH4654. We also included strains that expressed a truncated form of SasG such as those of USA300 strain LAC and USA100 strain N315. Together, these strains represent different subtypes of SasG that have been recently described (49). Finally, we included strains lacking a copy of the *sasG* gene altogether, such as USA200 strains MN8 and MRSA252, as controls for comparison. Strains with a functional, full-length version of SasG protein exhibited high levels of saliva-induced aggregation in the absence of *mgrA* (Fig. 1E) and we observed abundant SasG in cell wall preparations (Fig. 1F). The CF clinical isolate AH4654 exhibited lower expression levels and intermediate aggregation (Fig. 1E), although the genetic composition is almost identical to MW2, the functionality of the ArlRS-MgrA system in relation to SasG is not clear in this strain. Unexpectedly, Newman exhibited no visible expression of SasG protein (in WT or Δ*mgrA* mutant) and little aggregation despite having a full-length version of SasG encoded in the genome (Fig. 1E and F). N315 expressed a protein of size similar to SasG but did not clump at all. These data were confirmed by qPCR quantifying *sasG* expression (Fig. 1G). In general, our observations indicate that *S. aureus* strains with a full-length SasG, under conditions that induce *sasG* gene expression, will aggregate in the presence of human saliva.

### Molecular details of MgrA repression of *sasG* gene

**Fig 2:**
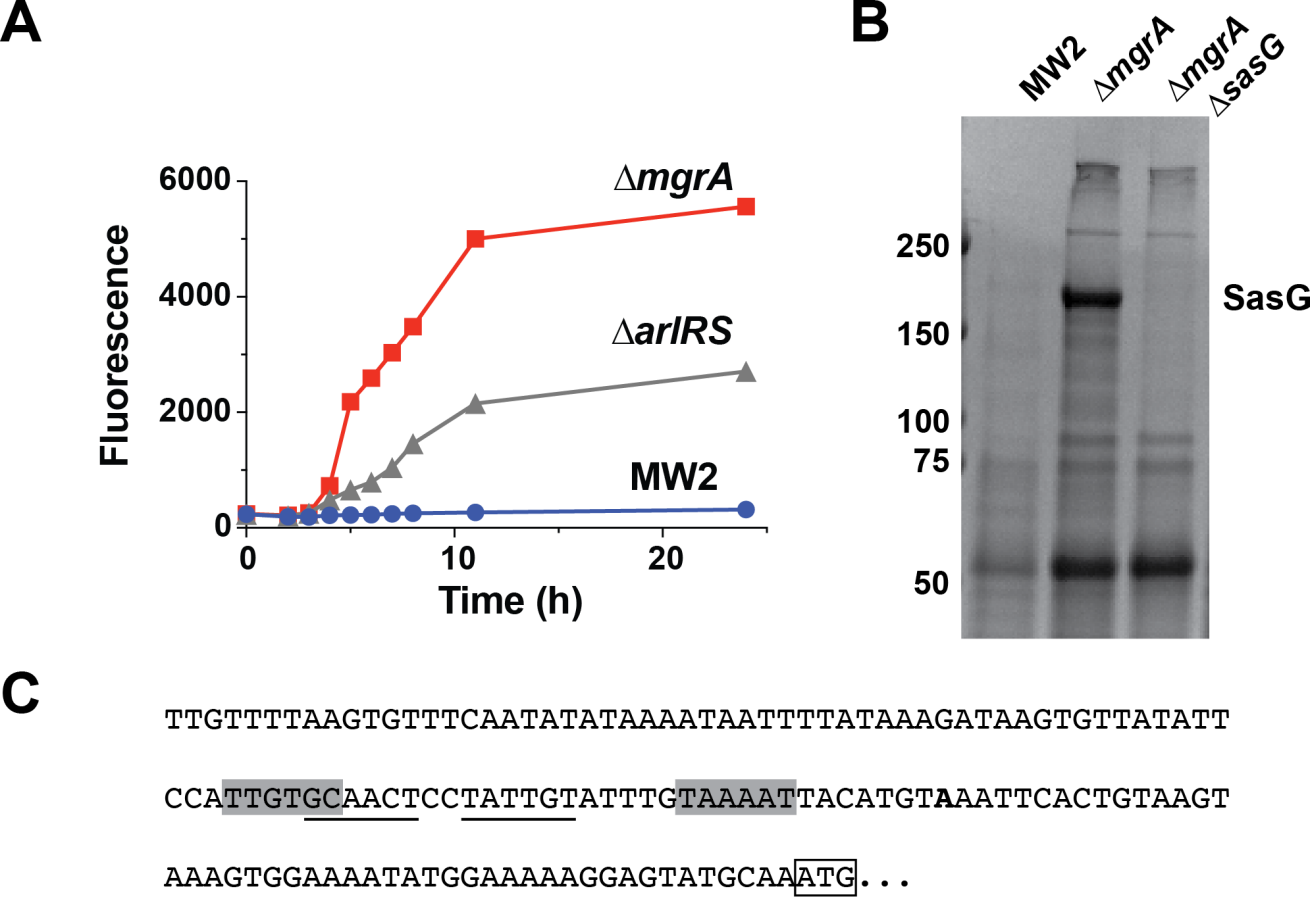
SasG expression is regulated by ArlRS and MgrA. (**A**) Expression of a P*_sasG_*-GFP transcriptional reporter in the wild-type strain MW2 and isogenic Δ*mgrA* and Δ*arlRS* mutants. (**B**) Coomassie-stained SDS-PAGE gel of shed surface proteins from MW2, as well as Δ*mgrA* and Δ*mgrA*Δ*sasG* mutants. The SasG band is indicated. (**C**) The transcription start site (in bold) of *sasG* was determined using 5′RACE. The ATG start codon is boxed, and putative −35 and −10 elements are shaded in gray. A potential MgrA binding site is underlined.

To investigate transcriptional control of *sasG* in the (CA-MRSA) USA400 strain MW2, we constructed a P*_sasG_*-sGFP reporter plasmid (pHC127) with *sasG* promoter fused to a gene encoding sGFP. This plasmid was transformed into mutants of the ArlRS and MgrA regulatory systems, previously suspected to repress the expression of SasG, and the expression levels were monitored over 24 h (Fig. 2A). The highest expression was observed in the Δ*mgrA* mutant, followed by the Δ*arlRS* mutant, with minimal expression in WT. The high expression in Δ*mgrA* mutant was confirmed at the protein level (Fig. 2B). We analyzed the *sasG* promoter region by 5′RACE to identify a putative housekeeping promoter and transcriptional start site (Fig. 2C). Putative MgrA repressor binding sites are shown that overlap the promoter region (48). Overall, our findings confirm that the expression of SasG in laboratory growth media is repressed by the ArlRS-MgrA regulatory cascade.

### SasG processing after A domain repeats promotes aggregation in human saliva

**Fig 3:**
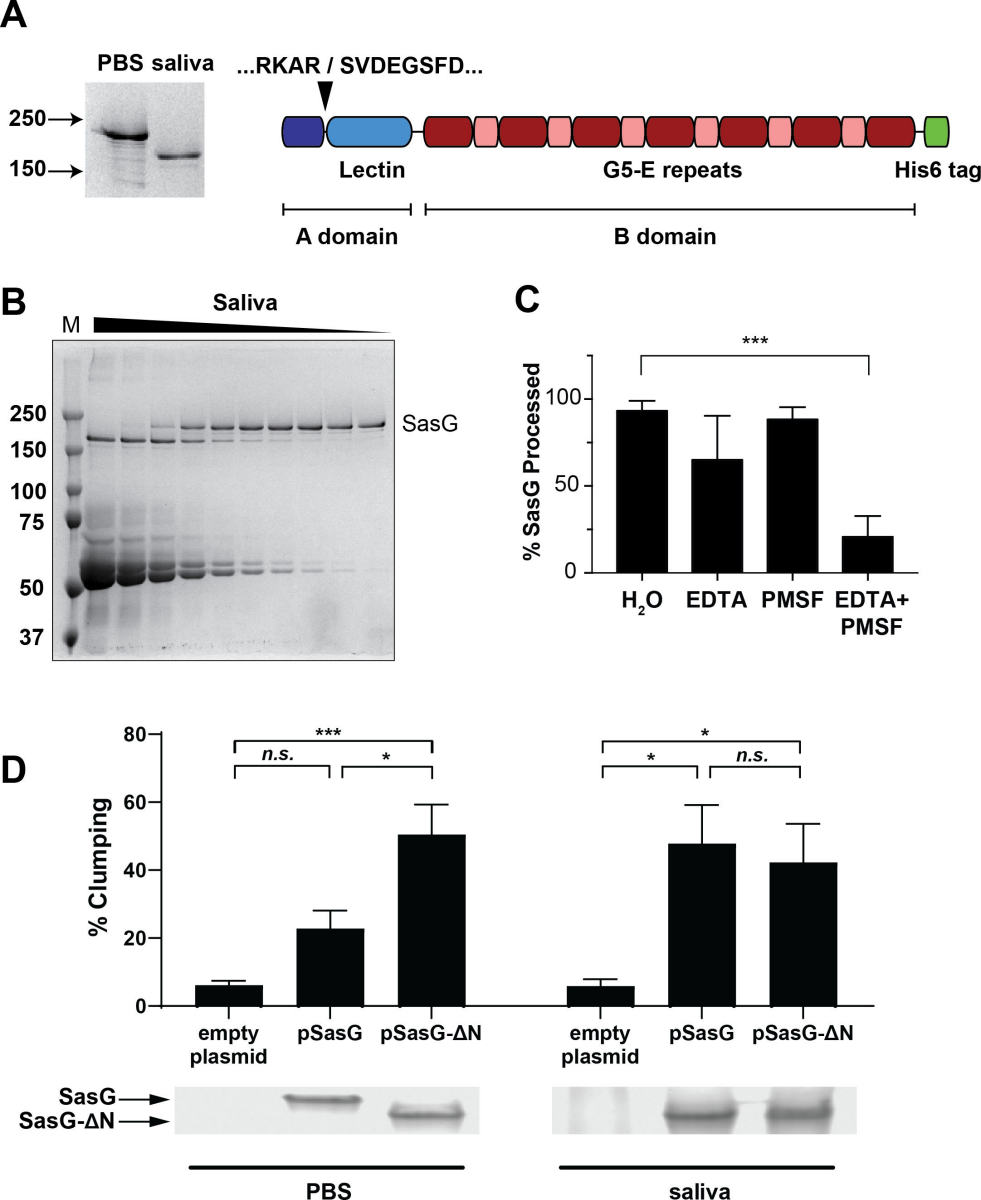
Saliva cleaves SasG within the A domain. (**A**) The *sasG* gene from *S. aureus* MW2 was cloned with a C-terminal His_6_ tag in place of the cell wall anchor, allowing it to be purified from *S. aureus* culture supernatants. This purified, full-length SasG was then incubated with human saliva for 1.5 h, resulting in SasG cleavage (shown in Coomassie-stained gel on the left). Cleaved SasG was re-purified and subjected to N-terminal sequencing, which showed the cleavage site to be N-terminal to the lectin domain. (**B**) Human saliva was concentrated ~5-fold before generating a 2-fold dilution series. Purified SasG was then added, and the reactions were incubated for 1 h at 37°C. (**C**) Saliva was pre-incubated with either 2.5 mM EDTA, 2.5 mM PMSF, or both, before adding purified SasG. Reactions were incubated for 2 h at 37°C before resolving on an SDS-PAGE gel. SasG bands were quantified, and the percentage processed to the shorter product was calculated. Results are averages of three experiments, with statistical significance calculated by ANOVA. ***, *P* < 0.001. (**D**) Aggregation of LAC strain, lacking its own SasG, and expressing from a plasmid either a full-length SasG construct or SasG construct with truncated N-terminal domain which replicates the effect of saliva processing. Aggregation was measured on *S. aureus* from overnight cultures suspended in saliva or PBS buffer for 1 h. *N* = 7. Coomassie-stained SDS-PAGE gels showing expression and processing of SasG constructs in each strain were prepared from cell wall preparations of the above-mentioned samples after the incubation.

As noted in Fig. 1, a large protein consistent with the size of SasG was upregulated in the Δ*mgrA* mutant and processed to a smaller version after incubation with human saliva (Fig. 1C and 3A). These observations suggest that proteases present in saliva could process SasG to smaller sizes. A previous report suggested that SasG possessed self-processing capability and that this cleavage occurred at multiple sites within the B domain (38). While the self-processing might be occurring in other experimental conditions, we did not observe background processing in our experiments when bacteria were incubated in PBS (Fig. 1C). By contrast, our results indicate that SasG may be processed by a host protease(s), and there may be a single cleavage site near one end of the protein, similar to what is seen with Aap (46).

To determine the location of the cleavage site within SasG, we cloned and purified the extracellular portion of SasG. The LPXTG cell wall anchor was replaced with a hexahistidine tag, and the protein was expressed in a *S. aureus* strain that lacks secreted proteases (56). Purified SasG was incubated with saliva and then re-purified before N-terminal sequencing to determine the cleavage site. The results revealed a cut site after Arg-144, which falls between the A repeats and lectin subdomain (Fig. 3A). This is similar to one of the two reported cleavage locations in Aap (46), but it is somewhat surprising because the removal of the entire A domain was thought to be required for both Aap and SasG B domain homodimerization and subsequent aggregation (43, 46). The cleavage of SasG by saliva was found to be dose-dependent (Fig. 3B), suggesting the presence of specific cleaving protease(s) inside the saliva. Therefore, purified SasG was incubated with saliva and protease inhibitors to identify the responsible protease(s). Minimal inhibition was seen with EDTA or PMSF alone, but in combination, they almost completely inhibited cleavage of SasG (Fig. 3C). This result suggests that saliva contains at least two proteases, a metalloprotease and a serine protease, that process SasG and promote bacterial aggregation.

To test whether this truncated form of SasG could promote aggregation, we cloned both full-length and truncated versions of *sasG* and expressed them in strain USA300 LAC, which does not express a functional SasG on its own due to a frameshift mutation in its *sasG* gene (57). While expression of full-length SasG had only minimal effect on aggregation in buffer and required saliva to facilitate a full-scale aggregation, the truncated version of SasG facilitated aggregation in buffer alone (Fig. 3D). This confirmed that removal of the 94 N-terminal amino acids of the A repeat region is sufficient to allow SasG to dimerize and promote aggregation.

### Fractionation to identify host proteases processing SasG

**Fig 4:**
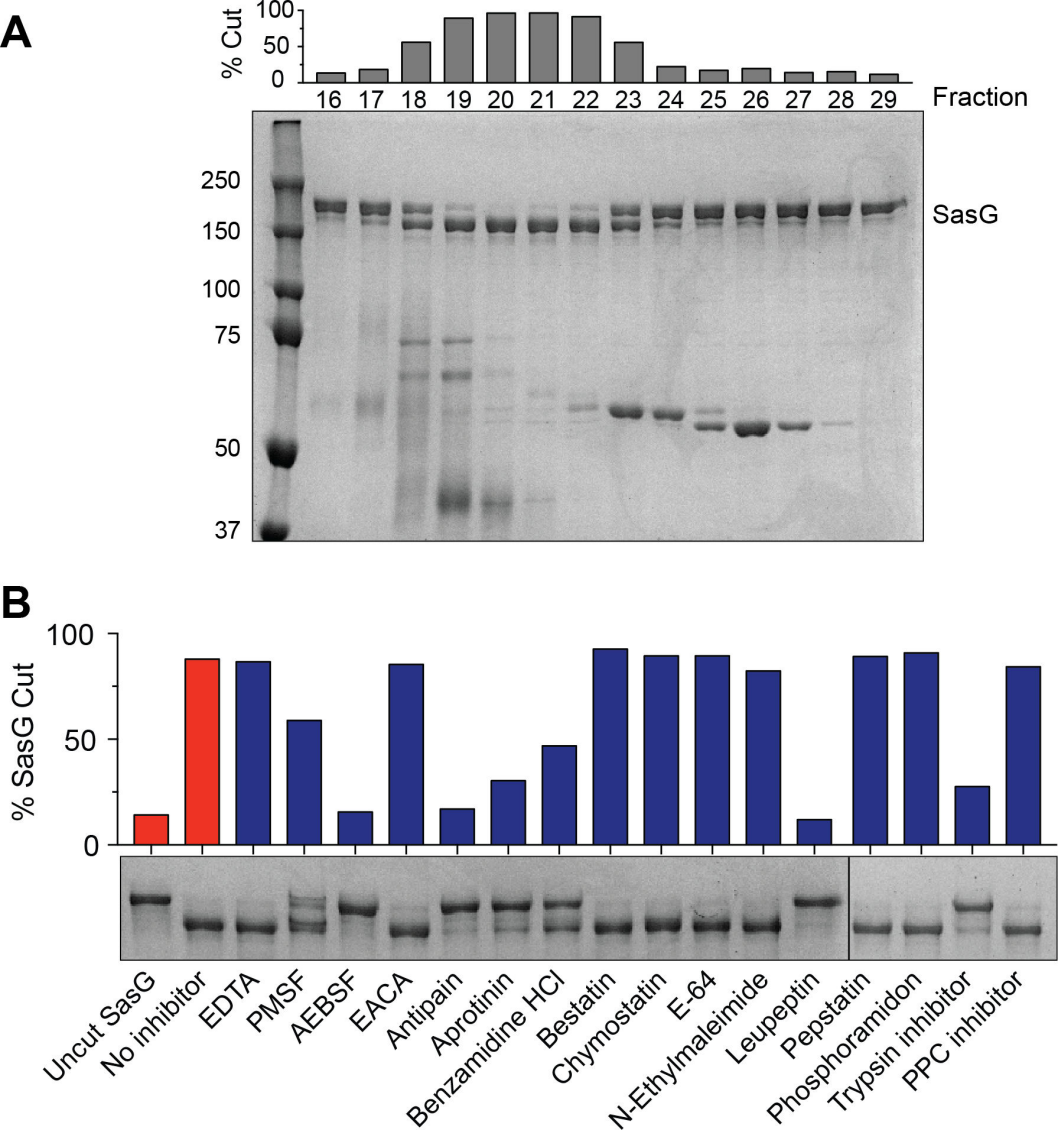
Partial purification of SasG processing enzyme from human saliva. (**A**) Pooled active fractions from passing saliva over an anion exchange column were then passed over a size exclusion column. Coomassie-stained gel shows SasG cleavage by selected fractions from the size exclusion purification. Fraction numbers are indicated above the gel, and bars show a percent SasG cleavage for each fraction. Molecular weight standards in kDa are indicated on the left. (**B**) Aliquots of fraction 20 were pre-incubated with the indicated protease inhibitors for 15 min before adding SasG. Cleavage of SasG was measured after 1.5 h at 37°C by separating on an SDS-PAGE gel and quantifying percent cleavage.

Clarified saliva was concentrated, filtered, and passed over multiple columns to separate the proteins into fractions. First, we used anion exchange chromatography followed by size exclusion chromatography. These fractions were then tested to see whether they could cleave purified SasG by running the reactions on SDS-PAGE gels and looking for a shift in SasG size (Fig. 4A). The level of SasG cleavage was highest in fractions 19–22 and these fractions were used going forward. In parallel, we tested the response of the isolated active saliva fraction with protease inhibitors to determine the exact class of the enzyme. The most inhibition was observed with AEBSF, antipain, and leupeptin, suggesting the enzyme present in the active fractions is a serine protease (Fig. 4B). After electrophoresis separation of fractions with the highest activity (Fig. 4A), individual bands were extracted from the gel and the protein(s) identified by MALDI mass spectrometry. Seven proteases were detected in these bands with significant peptide coverage, including trypsin-1, prostasin, serine protease 27, and various cathepsins (Table S1). Considering the protease inhibitor patterns (Fig. 4B), the best hit from the proteomics assessment was human trypsin.

### Validation of identified proteases

**Fig 5:**
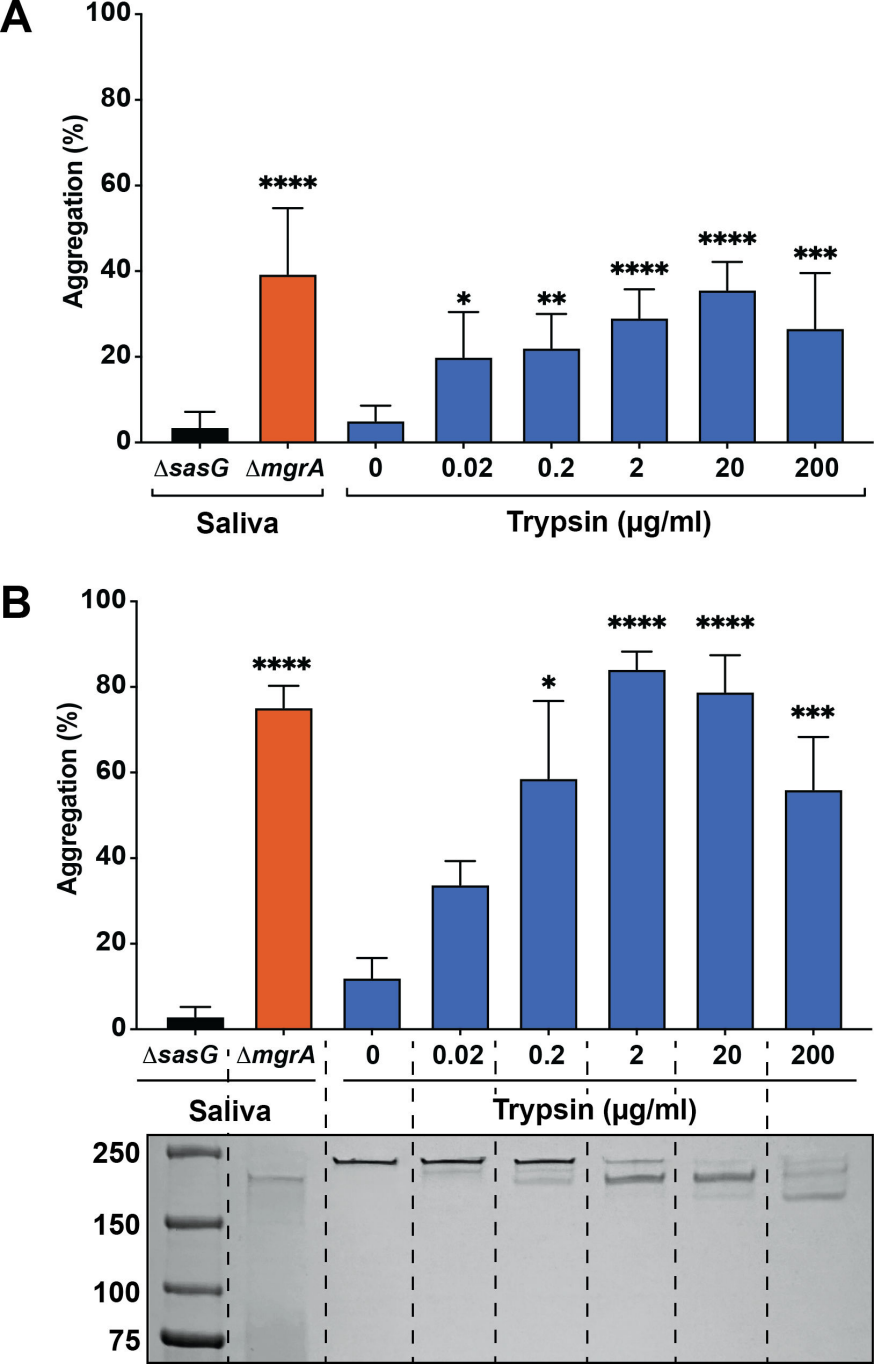
Trypsin can process SasG and promote *S. aureus* aggregation. *S. aureus* (**A**) 502a or (**B**) MW2 *mgrA* and *sasG* mutant strains were resuspended in either saliva or PBS supplemented with recombinant human trypsin and allowed to aggregate for 1 h. (**C**) Purified full-length SasG was incubated for 1 h with either human saliva or serial dilutions of trypsin before running on an SDS-PAGE gel and staining with Coomassie. Measurements are averages and standard deviations of three separate experiments. Significance was calculated by one-way ANOVA ****P < 0.0001.

We used commercially available human trypsin to test SasG processing and promotion of *S. aureus* aggregation. We performed aggregation assays of *S. aureus* strains MW2 and 502a with a range of recombinant human trypsin concentrations (0.02–200 μg/mL) (Fig. 5A and B). At the same concentrations, human trypsin was incubated with purified SasG, and dose-dependent SasG processing was observed via SDS-PAGE (Fig. 5C). Starting at 0.02 µg/mL, we observed cleavage of SasG, which correlated with an increase in aggregation. The levels of SasG cleavage and aggregation increased in a dose-dependent manner, reaching a peak at 2 µg/mL and decreasing slightly up to 20 µg/mL (Fig. 5A and B). These findings demonstrated that recombinant human trypsin can recapitulate the phenotype of SasG processing and *S. aureus* aggregation seen with saliva. At 200 µg/mL human trypsin, aggregation began decreasing due to degradation of the SasG protein (Fig. 5B).

### Role of SasG in pneumonia model

**Fig 6:**
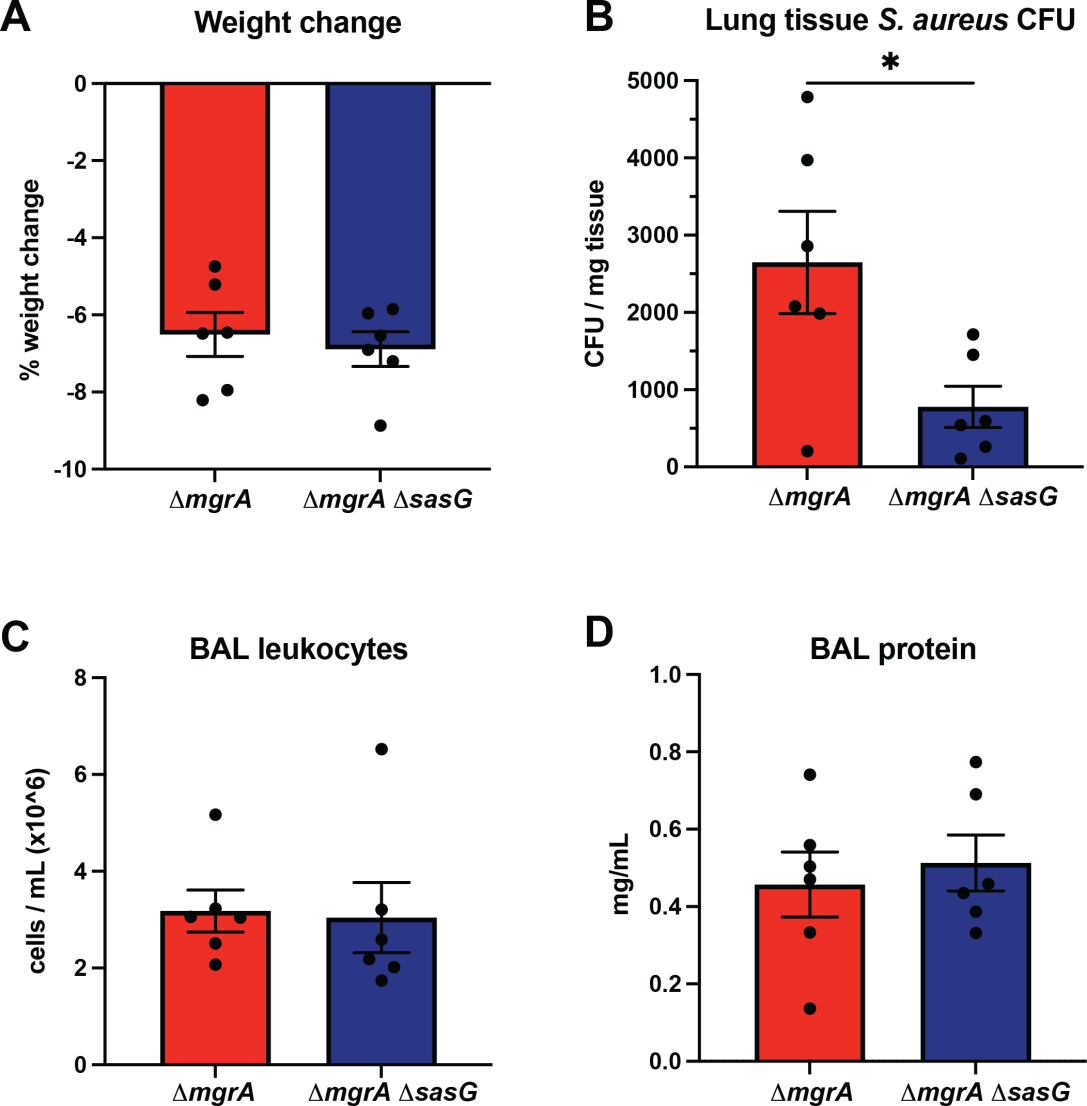
SasG is involved in *S. aureus* virulence in lung infection. Mice were infected intratracheally by *S. aureus* MW2 Δ*mgrA* and by its congenic strain Δ*mgrA* Δ*sasG* lacking SasG, and severity of pneumonia was assessed by weight loss (**A**), counting the CFU burden in lung homogenates (**B**), lung leukocyte recruitment in bronchoalveolar lavage (**C**), and protein infiltration in lavage fluid (**D**) after 24 h. The results were presented as means ± SEM, with statistical significance calculated by the Mann-Whitney test. *, *P* < 0.05.

To examine the biological relevance of SasG *in vivo*, we intratracheally infected mice with MW2 Δ*mgrA* (thus, SasG-expressing) or with Δ*mgrA* Δ*sasG* double mutant (Fig. 6). No evidence of systemic dissemination was observed in this model (Fig. 6A). The mice that were infected with the double mutant lacking SasG showed decreased number of colonies in the lungs (Fig. 6B), compared to the Δ*mgrA* strain expressing SasG. At the same time, markers of inflammation and tissue damage, that is the number of leukocytes (Fig. 6C) and level of protein (Fig. 6D) in the bronchoalveolar lavage (BAL), remain similar irrespective of the injected strain. The same trend of decreased bacterial counts and not significantly affected leukocytes and protein levels was also observed when a lower dose of *S. aureus* was used for infection (Fig. S1A through C). To validate our *in vivo* findings, we repeated our *in vitro* aggregation and SasG cleavage experiments with commercially available mouse proteases, airway trypsin-like protease (TMPRSS11D), and Kallikrein 1 (KLKB1), which are serine proteases found in the airways and function similarly to human trypsin (58–61) (Fig. S2 and S3).

We performed aggregation assays and SasG cleavage experiments as in Fig. 5 using murine proteases at a concentration range of 0.002–2 µg/mL. We observed that both murine proteases TMPRSS11D and KLKB1 could cleave SasG and induce aggregation at a concentration of 2 µg/mL (Fig. S2 and S3). Notably, TMPRSS11D, which is an ortholog to the corresponding human trypsin-like protease, induced significant *S. aureus* aggregation similar to both saliva and what was observed at the same concentration with human trypsin (Fig. S2). Overall, this suggests that during lung infection, the presence of SasG on the *S. aureus* surface has no effect on host response or local damage, but it does the benefit survival of the pathogen when faced with host immune response. Overall, the mouse pneumonia data indicate that the presence of SasG contributes to *S. aureus* virulence *in vivo*.

## DISCUSSION

Roughly one-third to half of healthy individuals are colonized by *S. aureus* in the nasal cavity and/or nasopharynx (62–64). While *S. aureus* colonization is benign in healthy adults, the presence of *S. aureus* in the respiratory tract is the major risk factor for developing pneumonia in the intensive care unit (65, 66). Despite the high rate of *S. aureus* carriage in the oral cavity, only preliminary studies have been performed on *S. aureus* interactions with human saliva proteins (67, 68). *S. aureus* predominantly binds human proteins using microbial surface components recognizing adhesive matrix molecules (MSCRAMMs) (69). We have previously shown that the ArlRS/MgrA regulatory cascade controls the expression of MSCRAMMs and other surface proteins that function in adhesion and immune evasion (48). Strains lacking either *arlRS* or *mgrA* overexpress these surface proteins, and in this work, we made the surprising discovery that a *S. aureus mgrA* mutant aggregates in the presence of human saliva. We found that intercellular aggregation is dependent on the expression of SasG but also requires host factors in saliva to process SasG.

**Fig 7:**
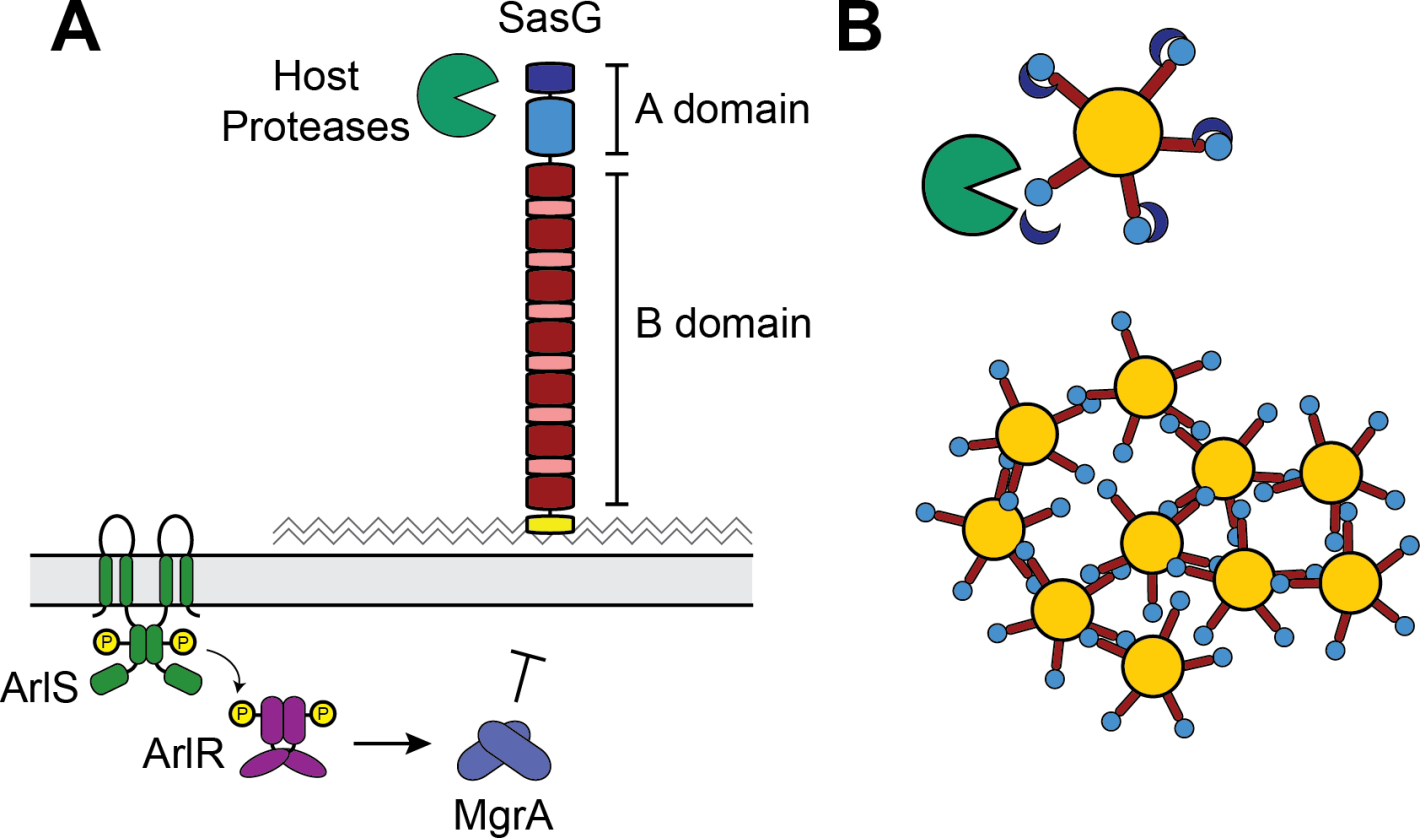
Model of SasG transcriptional and post-translational regulation. (**A**) The expression of *sasG* is repressed by the ArlRS-MgrA regulatory cascade. At the post-translational level, host proteases such as trypsin can remove the N-terminal end of the A domain. (**B**) Removal of the end of the A domain (dark blue) allows SasG to oligomerize with SasG molecules on neighboring cells, resulting in aggregation of *S. aureus*.

In previous studies, we demonstrated that full-length SasG is sufficient to block clumping and adhesion of cells by physically interfering with other surface proteins’ ability to bind to host matrix components (48, 51, 70, 71). However, SasG expression is low in *S. aureus* laboratory strains under standard *in vitro* conditions, which masks these clumping interference and aggregation phenotypes. Through our mapping of the *sasG* promoter and transcriptional reporter assay, we show that *sasG* expression is repressed by ArlRS/MgrA, and we identify a potential MgrA-binding site that overlaps with the *sasG* promoter. Therefore, inactivation of the ArlRS-MgrA cascade allows for high expression levels of *sasG*(Fig. 7A) .

We also found that there is significant variation in *sasG* expression and molecular characteristics among strains. It was recently reported that most *S. aureus* strains have a full-length copy of SasG (49), but not all express SasG at detectable levels (57) and some strains have truncated copies of SasG. USA400 MW2 and 502a encode full-length, surface-attached copies of SasG with 5 B-repeats which aggregate with high efficiency. Bioinformatic analysis of the CF isolate AH4654 genome revealed the *sasG*, *mgrA*, and *arlRS* genes, and their respective promoter regions, are all essentially identical to MW2. Interestingly, this CF isolate expresses SasG and aggregates natively (Fig. 1), similar to other *S. aureus* isolates that fall into the ST15/CC15 grouping (39). By contrast, strain Newman, despite encoding a full-length SasG, does not present it on its surface and does not aggregate with or without MgrA. The reason SasG is not functional in Newman is unclear at this time. Strains such as USA300 LAC and N315 have truncated copies of SasG due to frameshift mutations and therefore cannot aggregate (57). Other strains like MN8 and MRSA252 do not possess *sasG* and any observed aggregation was likely due to another surface protein.

SasG is one of the key drivers of biofilm formation in *S. aureus* (38–40, 45). SasG and its *S. epidermidis* homolog Aap consist of multiple domains with distinct functions (Fig. 1A). In *S. epidermidis,* the Aap A domain is known to be removed by the secreted metalloprotease SepA to facilitate biofilm accumulation (46), but native *S. aureus*-secreted proteases have not been found to cleave SasG in the same manner (38). Previous studies on *S. epidermidis* Aap also showed that exogenously added host proteases, such as trypsin and cathepsin G, could cleave Aap and enhance biofilm formation through processing (43). Our studies have found a parallel role for host proteases in cleaving *S. aureus* SasG and triggering aggregation (Fig. 7).

During infection, *S. aureus* uses mechanisms of aggregation and biofilm formation as the survival strategy to protect itself long term in response to environmental stressors, such as antimicrobials or host immune factors. *S. aureus* is a frequent colonizer of the oral cavity and can be isolated from dental plaques, periodontal pockets, and saliva, which represents a significant reservoir for opportunistic respiratory infections like aspiration pneumonia (55). It has been reported that up to 24%–36% of healthy adults harbor *S. aureus* in the oral cavity, with a higher incidence in individuals who are immunocompromised or undergoing antibiotic treatment (55, 68, 72). Oral *S. aureus* has been found to interact with and bind several salivary components, which is associated with inducing adhesion and aggregation mechanisms (67, 68, 73). Oral colonization and poor immune clearance of aggregated *S. aureus* present in saliva*,* particularly in immunocompromised individuals, are major risk factors for the development of lung infections following saliva aspiration (53, 55, 68, 72, 73). Our data demonstrate that the upregulation of *sasG* is associated with increased aggregation upon interaction with human saliva, which is known to contain numerous proteases (74). Considering that the aspiration of saliva secretions is a common precursor to lung infection (75), our findings indicate that salivary proteases are capable of cleaving SasG at a single site within the A domain. This processing removed the 94 amino acids that compose the A repeats, exposing the lectin and B domains to interact with neighboring cells and homodimerize (Fig. 7B). We fractionated the proteases to identify human trypsin and validated them with both commercially available human trypsin and murine salivary proteases. However, additional serine and metalloproteases may also contribute to the processing of SasG. From an adaptive standpoint, *S. aureus* may have evolved a surface protein like SasG that is proteolytically labile, which can sense environmental conditions and facilitate aggregation to protect *S. aureus* under stress.

Despite significant biochemical and structural studies on SasG, accompanied by experiments *in vitro*, no prior studies have determined its contribution to virulence in animal models of infection. However, simultaneous deletion of SasG and Eap did reduce insect mortality in a silkworm infection model (76). In this work, we now provide evidence that SasG contributes to *S. aureus* in the establishment of a lung infection. We demonstrated that SasG is important for *S. aureus* to survive and proliferate at the infection site and that interaction with host saliva in conjunction with aspiration may promote *S. aureus* survival by inducing aggregation. However, the presence of SasG did not impact the host response or damage to the host, suggesting it is solely important for *S. aureus* survival in a stressful environment.

In summary, we have shown that the global regulator MgrA controls the expression of the surface protein SasG. There is variation in the type and amount of SasG expressed among *S. aureus* strains, but expression of full-length SasG is associated with increased aggregation which is dependent on the presence of host proteases. We identified the serine protease human trypsin as a component of saliva that can process the SasG A domain to trigger aggregation (Fig. 7). Finally, we showed that SasG is important for full virulence in a *S. aureus* lung infection.

## MATERIALS AND METHODS

### Reagents and growth conditions

*S. aureus* strains and plasmids used in this work are listed in Table 1. AH4654 is one of 75 clinical isolates, isolated from 10 pediatric CF patients and kindly gifted by the Starner Lab, University of Iowa. *S. aureus* was cultured in tryptic soy broth (TSB) or brain heart infusion (BHI) broth, and *E. coli* was cultured in lysogeny broth (LB) at 37°C with shaking at 200 rpm. Antibiotics were added to the media at the following concentrations: chloramphenicol (Cam), 10 µg/mL; erythromycin (Erm), 5 µg/mL; and tetracycline (Tet), 1 µg/mL. *E. coli* strains with plasmids were maintained on media supplemented with ampicillin at 100 µg/mL; kanamycin at 50 µg/mL; or spectinomycin at 50 µg/mL. Porcine trypsin and the Protease Inhibitors Set (Roche) were purchased from Sigma. Recombinant proteases (human trypsin, mouse kallikrein 1, and airway trypsin-like protease) were purchased from G-Biosciences, Sigma-Aldrich, and ThermoFisher Scientific (Biotechne). Stimulated saliva was collected over 10–30 min by chewing on paraffin wax. Particulate material was removed by centrifugation, and this clarified saliva was stored at 4°C for up to 2 days.

**TABLE 1:** Bacterial strains and plasmids

Strain or plasmid	Genotype or properties	Reference or source
*E. coli*		
DH5α	Cloning strain	Protein Express
DC10B	Cloning strain (*dcm*^-^)	(77)
T7 Express	Protein expression strain	NEB
*S. aureus*		
RN4220	Restriction-deficient cloning host	(78)
MW2	USA400 CA-MRSA	(79)
AH3422	MW2 Δ*mgrA*	(48)
AH3989	MW2 Δ*mgrA* Δ*sasG*	(48)
AH1263	USA300 CA-MRSA Erm^S^ (LAC*)	(80)
AH3375	LAC Δ*mgrA*	(48)
AH1919	LAC* Δ*aur* Δ*sspAB* ΔstaphopainA Δ*spl*::erm	(56)
AH4607	LAC* Δ*aur* Δ*sspAB* ΔstaphopainA Δ*spl*::erm φ11att::*tet*	This work
502a	ST5 MSSA	
AH3625	502a Δ*mgrA::*tetM	(48)
Newman	MSSA	(81)
AH3472	Newman Δ*mgrA::*tetM	(48)
N315	USA100 MRSA	(82)
AH3473	N315 Δ*mgrA::*tetM	(48)
MN8	USA200 MSSA	(83)
AH3480	MN8 Δ*mgrA::*tetM	(48)
MRSA252	USA200 HA-MRSA	(84)
AH3483	MRSA252 Δ*mgrA::*tetM	(48)
AH4654	MSSA CF Isolate	This work
AH4728	AH4654 Δ*sasG*::Tn Erm	This work
Plasmids		
pALC2073	Tetracycline-inducible shuttle vector, Cam^R^	(85)
pRMC2	Tetracycline-inducible shuttle vector, Cam^R^	(86)
pCM28	Empty vector control for pCM29, Cam^R^	(80)
pCM29	sGFP expression vector, Cam^R^	(87)
pTEV5	Expression vector with TEV-cleavable His6 tag, Amp^R^	(88)
pHC66	*mgrA* complementation vector, Cam^R^	(48)
pHC89	pALC2073-*sasG*	This work
pHC90	pALC2073-*sasG*-His6 (secreted)	This work
pHC108	pTEV5 *sasG* B repeat	This work
pHC116	pALC2073-*sasG* ΔN	This work
pHC127	P*_sasG_*-sGFP, Cam^R^	This work

### Recombinant DNA and genetic techniques

*E. coli* DH5α and DC10B were used as cloning hosts for plasmid construction. Restriction enzymes, DNA ligase, and Phusion DNA polymerase were purchased from New England Biolabs. The plasmid mini-prep and gel extraction kits were purchased from Invitrogen. *S. aureus* genomic DNA was purified using the Puregene yeast/bacteria kit B (Qiagen). Lysostaphin, used for *S. aureus* DNA extractions, was purchased from Sigma. Plasmids were purified from *S. aureus* RN4220 or *E. coli* DC10B and electroporated into MRSA LAC strains as described previously (77, 89). Bacteriophage transductions between *S. aureus* strains were performed with phage 11 as described previously (90). All oligonucleotides were ordered from IDT (Coralville, IA) and are listed in Table 2. Routine DNA sequencing was performed at the University of Iowa DNA Core Facility or the Molecular Biology Service Center at the University of Colorado Anschutz Medical Campus. Whole-genome sequencing was performed at the University of Iowa DNA Core Facility with the Illumina MiSeq platform followed by *de novo* contig generation with the SPAdes genome assembler (91), and quality assessed with QUAST (92). Assemblies were annotated with Prokka (93).

**TABLE 2:** Primers

Code	Name	Sequence
HC233	sasG Tn (up) confirmation	ACTGTAAGCAAAGTGGAAAATATGG
HC233	sasG Tn(down) confirmation	CTCTGAACCTTTCAAGTCAGTTCTC
HC416	MW2 sasG 5′ KpnI	GTTGGTACCCACTGTAAGTAAAGTGGAAAATATGGAA
HC418	MW2 sasG His 3′ SacI	GTTGAGCTCTTAATGATGATGATGATGATGACCTTCTGCTCGTTTTTTCTCTTGAT
HC598	PsasG 5′XbaI	GAAGTTCTAGAAGTATGTTTCGAGATTTTAATATCTTGG
HC599	PsasG 3′KpnI	GTTGAGGTACCCTTTTTCCATATTTTCCACTTTACTTAC
HC608	QT	CCAGTGAGCAGAGTGACGAGGACTCGAGCTCAAGCTTTTTTTTTTTTTTTTT
HC609	QO	CCAGTGAGCAGAGTGACG
HC610	QI	GAGGACTCGAGCTCAAGC
HC611	sasG GSP-RT	TGGACATTATCTTTTAATGTAGTTGGATTCTC
HC612	sasG GSP1	AGTTCCCAAATACATTAGTGAGCC
HC613	sasG GSP2	TAGATGCTGTTCCAACTGTAAATTTTC
KK015	sasG RT-qPCR fwd	GCAGAAGCAGCTGAAAACAA
KK016	sasG RT-qPCR rev	GTGGTGCAGTGTCTTTGTTTG
KK23	gyrB RT-qPCR fwd	AACGGACGTGGTATCCCAGTTGAT
KK24	gyrB RT-qPCR rev	CCGCCAAATTTACCACCAGCATGT

### RNA purification and RT-qPCR

Bacterial cultures were grown overnight in TSB and then subcultured to an OD_600_ of 1.5. Cells were then pelleted and washed with RNAprotect Bacterial Reagent (Qiagen). To extract RNA, cells were lysed with lysostaphin for 30 min at room temperature, and RNA was purified using the RNeasy Mini Kit (Qiagen). Following RNA purification, genomic DNA was then removed using the Turbo DNase Kit (Ambion). cDNA was generated from a DNase-treated RNA template using the iScript cDNA synthesis kit (Bio-Rad). To perform quantitative PCR (qPCR), Primers KK15 and KK16 were used for *sasG*, and KK23 and KK24 for DNA gyrase (*gyrB*), as described previously (48). qPCR was performed by amplifying cDNA in 20 µL reaction volumes with iTaq Universal SYBR Green Supermix (Bio-Rad) in the CFX96 Touch Real-Time PCR System (Bio-Rad) under the following conditions: 3 min at 95°C, 40 cycles of 10 s at 95°C, and 30 s at 59°C, followed by a dissociation curve. No template and no reverse transcription controls were performed in parallel. Experiments were performed in biological triplicate with two technical replicates, and expression was normalized to *gyrB*.

### *sasG* promoter mapping and GFP fusion plasmid

The *sasG* promoter was mapped using rapid amplification of 5′ cDNA ends (5′ RACE) (94). Template RNA was purified from MW2 Δ*mgrA* using the RNeasy Mini Kit (Qiagen) as previously described (48). Primers used were the general 5′ RACE primers (94) HC608, HC609, and HC610, and the *sasG*-specific primers HC611, HC612, and HC613. To generate the P*_sasG_*-GFP fusion plasmid, the region upstream of *sasG* was amplified using primers HC598 and HC599. The fragment was digested using XbaI and KpnI before ligating into pCM29 (87). The resulting plasmid, pHC127, encodes the *sasG* promoter upstream of an optimized ribosome binding site and codon-optimized gene for superfolder GFP. To assess expression, overnight cultures were diluted 1:100 in TSB containing chloramphenicol in a black 96-well plate. Plates were incubated at 37°C with shaking in a humidified microtiter plate shaker (Stuart). A Tecan Infinite M200 plate reader was used to periodically measure OD_600_ and fluorescence intensity with excitation at 495 nm and emission at 515 nm. Values represent averages and standard deviations of triplicate wells.

### *S. aureus* aggregation assay

*S. aureus* cultures (5 mL) were grown overnight in TSB with shaking at 37°C. One milliliter of culture was harvested by centrifugation and the media was discarded. Saliva was collected and clarified by centrifugation and filtration. Recombinant human trypsin was activated by incubating 0.4 mg/mL with 0.2 µg/mL porcine trypsin for 1 h. Following activation, the recombinant trypsin was diluted in phosphate-buffered saline to the indicated concentrations. The cells were resuspended in 1 mL of either phosphate-buffered saline, clarified human saliva, or activated recombinant human trypsin solution. Tubes were allowed to sit for 1 h at room temperature, and then aggregation was visually assessed. For quantification of aggregation, 100 µL of liquid was removed from the top of the tube at 0 h and 1 h, and the optical density at 600 nm was measured in a 96-well plate in a Tecan infinite M200 plate reader. Measurements represent averages and standard deviations of experiments performed on three separate days.

To assess *S. aureus* aggregation induced by the recombinant mouse kallikrein one and airway trypsin-like proteases, *S. aureus* cultures were prepared as described above. Recombinant proteases were prepared and/or activated according to the manufacturer’s guidelines to a final concentration of 100 µg/mL. Proteases were then diluted in phosphate-buffered saline to the indicated reaction concentrations. Due to volume limitations of the recombinant proteases, cells were then resuspended in 500 µL of either clarified human saliva, phosphate-buffered saline, or recombinant protease solutions and incubated for 1 h at room temperature. Aggregation was quantified as described above.

### Cell wall preparations

For the preparation of cell wall proteins after aggregation assays, the tubes were centrifuged, and the cells were washed twice with PBS. The cells were resuspended in 500 µL of protoplasting buffer (10 mM Tris pH 8, 10 mM MgSO_4_, 30% raffinose). Lysostaphin (25 µg) was added and the cells were incubated for 1 h at 37°C. The tubes were centrifuged for 3 min at max speed, and 500 µL of supernatant was transferred to a new tube. Proteins were precipitated by adding 125 µL of cold trichloroacetic acid and leaving it on ice for 2 h. Precipitated proteins were pelleted by centrifuging at max speed for 10 min. The pellet was washed twice with 500 µL of cold 100% ethanol and then inverted to dry. The pellets were resuspended in 36 µL of SDS-PAGE loading dye, heated to 85°C, and then 10 µL was loaded on a 7.5% acrylamide gel.

### Purification of full-length SasG

The *sasG* gene from *S. aureus* MW2 was amplified using primers HC416 and HC418 (Table 2), which remove the last 33 amino acids of SasG, including the LPXTG cell wall anchor, and replace them with a glycine followed by six histidine residues. This C-terminally tagged, secreted version of *sasG* was cloned into pALC2073 under the control of an anhydrotetracycline-inducible promoter, generating pHC90. We decided to purify this version of SasG from *S. aureus* LAC, which does not have an intact copy of *sasG* on the chromosome. To avoid potential proteolysis, we used a previously developed strain of LAC lacking secreted proteases (AH1919). In addition, we modified AH1919 to be resistant to anhydrotetracycline by integrating the empty vector pLL29 (95) in the phage 11 attachment site, generating host strain AH4607.

For expression of SasG, pHC90 was moved into AH4607 and a 5 mL culture was grown overnight at 37°C in TSB with chloramphenicol. This overnight culture was used to inoculate 1 L of TSB supplemented with chloramphenicol and 0.15 µg/mL anhydrotetracycline. The culture was grown with shaking for ~6.5 h at 37°C. Cells were removed by centrifugation, and the culture supernatant was concentrated to ~30 mL using an Amicon stirring pressure concentrator with a 100 kDa cutoff filter. The supernatant was dialyzed twice against binding buffer (50 mM sodium phosphate, 300 mM NaCl, pH 8). SasG-His6 was then purified using a pre-packed 5 mL IMAC cartridge (Bio-Rad) on a Bio-Rad FPLC. SasG-His6 was eluted with a linear gradient up to 100% elute buffer (50 mM sodium phosphate, 300 mM NaCl, 250 mM imidazole, pH 8). The protein was then concentrated and dialyzed against the storage buffer (20 mM sodium phosphate, 150 mM NaCl, pH 7.5). Glycerol was added to 20% before flash freezing and storing at −80°C.

### SasG processing assays

Purified, full-length SasG was diluted 10-fold in phosphate-buffered saline, and 2 µL of this dilution was combined with 2 µL of water and 16 µL of clarified saliva or saliva fraction. Dilutions of recombinant human trypsin and murine proteases were prepared as described above. 2 µL of SasG was added to 18 µL of recombinant human trypsin solution to initiate the reaction. Reactions were incubated for 1–3 h at 37°C as indicated. Processing was then quenched by adding 10 µL of 2× Laemelli SDS-PAGE loading buffer (Bio-Rad) and heating to 65°C. 10 µL of this was loaded on a 7.5% or 10% gel, or a 4%–20% gradient gel. For calculating the percentage of SasG processed, Coomassie-stained gels were scanned and quantified using Image Studio Lite (LiCor).

### Identifying the cleavage site within SasG

A large SasG cleavage reaction was set up using 100 µL of purified SasG-His6, 900 µL of PBS, and 4 mL of clarified filtered saliva. The reaction was allowed to incubate for 1.5 h at 37°C. The solution was then exchanged to a binding buffer (same as above) using a 100 kDa molecular weight cutoff filter (Amicon). SasG-His was re-purified using HIS-Select resin (Sigma) and eluted with binding buffer containing increasing concentrations of imidazole. Fractions containing SasG-His were pooled and concentrated to ~0.5 mL, and 2, 4, 6, and 8 µL aliquots were mixed with SDS-PAGE buffer, boiled, and run on a 4%–15% gradient gel. Proteins were then transferred to a PVDF membrane using a Trans-blot Turbo transfer system (Bio-Rad) and the membrane was stained with Coomassie. N-terminal sequencing of cleaved SasG was carried out by Edman degradation using a Shimadzu PPSQ-53A Gradient Protein Sequencer at the Protein Facility at Iowa State University.

### Partial purification of proteases from human saliva

Stimulated saliva (~90 mL) was collected over 1 day and centrifuged at 30,000 × *g* to remove debris. Clarified saliva was filtered and then concentrated to ~3 mL using 30,000 MWCO centricon concentrators (Amicon) and dialyzed against buffer A (20 mM Tris pH 8, 2 mM NaCl). The sample was then separated by anion exchange chromatography using a HiScreen Capto Q column (GE Life Sciences), eluting with a linear gradient up to 100% buffer B (20 mM Tris pH 8, 1 M NaCl). Fractions were tested using the SasG processing assay described above, except that tubes were incubated for 2 h at 37°C before running on an SDS-PAGE gel. Active fractions were pooled, concentrated to ~350 µL, and loaded on an SEC70 size exclusion column (Bio-Rad). The running buffer consisted of 20 mM Tris pH 8 and 100 mM NaCl. 0.5 mL fractions were collected and tested for their ability to cleave SasG as described above, and a couple of fractions (20 and 21) was selected for further analysis. Protease inhibitors (Sigma) were used according to the manufacturer’s instructions. For protein identification, bands were excised from an SDS-PAGE gel and analyzed at the Proteomics Facility in the University of Iowa Carver College of Medicine.

### Pneumonia model

All mouse experiments were conducted in accordance with National Institutes of Health guidelines and previously approved by the University of Colorado Institutional Animal Care and Use Committee. Wild-type (WT) female BALB/c, 6–8 weeks old, were purchased from Jackson Laboratories (Bar Harbor, ME). Mice were anesthetized with isoflurane inhalation and challenged with approximately 2 × 10^8^ colony-forming units (CFU)/30 µL of either mutant (Δ*mgrA* or Δ*mgrA* Δ*sasG*) *S. aureus* MW2 strain intratracheally. A blunt-tipped, bent 18 g Hamilton syringe was used to administer 30 µL of *S. aureus* directly into the lungs. Mice were left to recover for 24 h after which were euthanized using a lethal dose of ketamine/xylazine. The trachea was cannulated and the right lobes were tied off allowing for unilateral bronchial alveolar lavage (BAL) fluid isolation from the left lung. The right lobes were weighed and then homogenized for CFU determination. As a measure of lung inflammation and injury, leukocytes and protein in BAL fluid were measured.

## Data Availability

The draft genome of AH4654 was deposited to NCBI and Illumina data are available in GenBank (accession no. JAPQKW000000000).

## SUPPLEMENTAL MATERIAL

Supplemental Figures mbio.03483-23-s0001.docx Figures S1-S3.

Supplemental Table mbio.03483-23-s0002.docx Table S1.

ASM does not own the copyrights to Supplemental Material that may be linked to, or accessed through, an article. The authors have granted ASM a non-exclusive, world-wide license to publish the Supplemental Material files. Please contact the corresponding author directly for reuse.

## References

[1] Mertz D, Frei R, Periat N, Zimmerli M, Battegay M, Flückiger U, Widmer AF. 2009. Exclusive Staphylococcus aureus throat carriage: at-risk populations. Arch Intern Med 169:172–178. doi:10.1001/archinternmed.2008.536 19171814

[2] Gorwitz RJ, Kruszon‐Moran D, McAllister SK, McQuillan G, McDougal LK, Fosheim GE, Jensen BJ, Killgore G, Tenover FC, Kuehnert MJ. 2008. Changes in the prevalence of nasal colonization with Staphylococcus aureus in the United States, 2001-2004. J Infect Dis 197:1226–1234. doi:10.1086/533494 18422434

[3] Miller LG, Diep BA. 2008. Clinical practice: colonization, fomites, and virulence: rethinking the pathogenesis of community-associated methicillin-resistant Staphylococcus aureus infection. Clin Infect Dis 46:752–760. doi:10.1086/526773 18220477

[4] Donkor ES, Kotey FC. 2020. Methicillin-resistant Staphylococcus aureus in the oral cavity: implications for antibiotic prophylaxis and surveillance. Infect Dis (Auckl) 13:1178633720976581. doi:10.1177/1178633720976581 33402829 PMC7739134

[5] Kearney A, Kinnevey P, Shore A, Earls M, Poovelikunnel TT, Brennan G, Humphreys H, Coleman DC. 2020. The oral cavity revealed as a significant reservoir of Staphylococcus aureus in an acute hospital by extensive patient, healthcare worker and environmental sampling. J Hosp Infect 105:389–396. doi:10.1016/j.jhin.2020.03.004 32151672

[6] Kluytmans JA, Mouton JW, Ijzerman EP, Vandenbroucke-Grauls CM, Maat AW, Wagenvoort JH, Verbrugh HA. 1995. Nasal carriage of Staphylococcus aureus as a major risk factor for wound infections after cardiac surgery. J Infect Dis 171:216–219. doi:10.1093/infdis/171.1.216 7798667

[7] Muñoz P, Hortal J, Giannella M, Barrio JM, Rodríguez-Créixems M, Pérez MJ, Rincón C, Bouza E. 2008. Nasal carriage of S. aureus increases the risk of surgical site infection after major heart surgery. J Hosp Infect 68:25–31. doi:10.1016/j.jhin.2007.08.010 17945393

[8] Wertheim HFL, Vos MC, Ott A, van Belkum A, Voss A, Kluytmans JAJW, van Keulen PHJ, Vandenbroucke-Grauls CMJE, Meester MHM, Verbrugh HA. 2004. Risk and outcome of nosocomial Staphylococcus aureus bacteraemia in nasal carriers versus non-carriers. Lancet 364:703–705. doi:10.1016/S0140-6736(04)16897-9 15325835

[9] von Eiff C, Becker K, Machka K, Stammer H, Peters G. 2001. Nasal carriage as a source of Staphylococcus aureus bacteremia. Study group. N Engl J Med 344:11–16. doi:10.1056/NEJM200101043440102 11136954

[10] Corne P, Marchandin H, Jonquet O, Campos J, Bañuls A-L. 2005. Molecular evidence that nasal carriage of Staphylococcus aureus plays a role in respiratory tract infections of critically ill patients. J Clin Microbiol 43:3491–3493. doi:10.1128/JCM.43.7.3491-3493.2005 16000487 PMC1169150

[11] Weiner LM, Webb AK, Limbago B, Dudeck MA, Patel J, Kallen AJ, Edwards JR, Sievert DM. 2016. Antimicrobial-resistant pathogens associated with healthcare-associated infections: summary of data reported to the national healthcare safety network at the centers for disease control and prevention, 2011-2014. Infect Control Hosp Epidemiol 37:1288–1301. doi:10.1017/ice.2016.174 27573805 PMC6857725

[12] Zimlichman E, Henderson D, Tamir O, Franz C, Song P, Yamin CK, Keohane C, Denham CR, Bates DW. 2013. Health care-associated infections: a meta-analysis of costs and financial impact on the US health care system. JAMA Intern Med 173:2039–2046. doi:10.1001/jamainternmed.2013.9763 23999949

[13] Cámara M, Green W, MacPhee CE, Rakowska PD, Raval R, Richardson MC, Slater-Jefferies J, Steventon K, Webb JS. 2022. Economic significance of biofilms: a multidisciplinary and cross-sectoral challenge. NPJ Biofilms Microbiomes 8:42. doi:10.1038/s41522-022-00306-y 35618743 PMC9135682

[14] Kavanagh KT. 2019. Control of MSSA and MRSA in the United States: protocols, policies, risk adjustment and excuses. Antimicrob Resist Infect Control 8:103. doi:10.1186/s13756-019-0550-2 31244994 PMC6582558

[15] Kourtis AP, Hatfield K, Baggs J, Mu Y, See I, Epson E, Nadle J, Kainer MA, Dumyati G, Petit S, Ray SM, Ham D, Capers C, Ewing H, Coffin N, McDonald LC, Jernigan J, Cardo D, Emerging Infections Program MRSA author group. 2019. Vital signs: epidemiology and recent trends in methicillin-resistant and in methicillin-susceptible Staphylococcus aureus bloodstream infections - United States. MMWR Morb Mortal Wkly Rep 68:214–219. doi:10.15585/mmwr.mm6809e1 30845118 PMC6421967

[16] Han A, Zenilman JM, Melendez JH, Shirtliff ME, Agostinho A, James G, Stewart PS, Mongodin EF, Rao D, Rickard AH, Lazarus GS. 2011. The importance of a multifaceted approach to characterizing the microbial flora of chronic wounds. Wound Repair Regen 19:532–541. doi:10.1111/j.1524-475X.2011.00720.x 22092791 PMC3227014

[17] Davies CE, Hill KE, Wilson MJ, Stephens P, Hill CM, Harding KG, Thomas DW. 2004. Use of 16S ribosomal DNA PCR and denaturing gradient gel electrophoresis for analysis of the microfloras of healing and nonhealing chronic venous leg ulcers. J Clin Microbiol 42:3549–3557. doi:10.1128/JCM.42.8.3549-3557.2004 15297496 PMC497624

[18] Gjødsbøl K, Christensen JJ, Karlsmark T, Jørgensen B, Klein BM, Krogfelt KA. 2006. Multiple bacterial species reside in chronic wounds: a longitudinal study. Int Wound J 3:225–231. doi:10.1111/j.1742-481X.2006.00159.x 16984578 PMC7951738

[19] Cystic Fibrosis Foundation. 2016. Patient registry, 2016 annual data report. Bethesda, Maryland, USA.

[20] Paharik AE, Horswill AR. 2016. The staphylococcal biofilm: adhesins, regulation, and host response. Microbiol Spectr 4. doi:10.1128/microbiolspec.VMBF-0022-2015 PMC4887152 27227309

[21] Donlan RM. 2001. Biofilms and device-associated infections. Emerg Infect Dis 7:277–281. doi:10.3201/eid0702.010226 11294723 PMC2631701

[22] Haaber J, Cohn MT, Frees D, Andersen TJ, Ingmer H. 2012. Planktonic aggregates of Staphylococcus aureus protect against common antibiotics. PLoS One 7:e41075. doi:10.1371/journal.pone.0041075 22815921 PMC3399816

[23] Fux CA, Wilson S, Stoodley P. 2004. Detachment characteristics and oxacillin resistance of Staphyloccocus aureus biofilm emboli in an in vitro catheter infection model. J Bacteriol 186:4486–4491. doi:10.1128/JB.186.14.4486-4491.2004 15231780 PMC438612

[24] Horst SA, Hoerr V, Beineke A, Kreis C, Tuchscherr L, Kalinka J, Lehne S, Schleicher I, Köhler G, Fuchs T, Raschke MJ, Rohde M, Peters G, Faber C, Löffler B, Medina E. 2012. A novel mouse model of Staphylococcus aureus chronic osteomyelitis that closely mimics the human infection: an integrated view of disease pathogenesis. Am J Pathol 181:1206–1214. doi:10.1016/j.ajpath.2012.07.005 22902429

[25] Fazli M, Bjarnsholt T, Kirketerp-Møller K, Jørgensen B, Andersen AS, Krogfelt KA, Givskov M, Tolker-Nielsen T. 2009. Nonrandom distribution of Pseudomonas aeruginosa and Staphylococcus aureus in chronic wounds. J Clin Microbiol 47:4084–4089. doi:10.1128/JCM.01395-09 19812273 PMC2786634

[26] Bjarnsholt T, Alhede M, Alhede M, Eickhardt-Sørensen SR, Moser C, Kühl M, Jensen PØ, Høiby N. 2013. The in vivo biofilm. Trends Microbiol 21:466–474. doi:10.1016/j.tim.2013.06.002 23827084

[27] DePas WH, Starwalt-Lee R, Van Sambeek L, Ravindra Kumar S, Gradinaru V, Newman DK. 2016. Exposing the three-dimensional biogeography and metabolic states of pathogens in cystic fibrosis sputum via hydrogel embedding, clearing, and rRNA labeling. mBio 7:e00796-16. doi:10.1128/mBio.00796-16 27677788 PMC5040109

[28] Kappler M, Nagel F, Feilcke M, Kröner C, Pawlita I, Naehrig S, Ripper J, Hengst M, von Both U, Forstner M, Hector A, Griese M. 2016. Eradication of methicillin resistant Staphylococcus aureus detected for the first time in cystic fibrosis: a single center observational study. Pediatr Pulmonol 51:1010–1019. doi:10.1002/ppul.23519 27378061

[29] Kiefer A, Bogdan C, Melichar VO. 2018. Melichar, successful eradication of newly acquired MRSA in six of seven patients with cystic fibrosis applying a short-term local and systemic antibiotic scheme. BMC Pulm Med 18:20. doi:10.1186/s12890-018-0588-6 29370836 PMC5785857

[30] Hall H, Gadhok R, Alshafi K, Bilton D, Simmonds NJ. 2015. Eradication of respiratory tract MRSA at a large adult cystic fibrosis centre. Respir Med 109:357–363. doi:10.1016/j.rmed.2015.01.013 25683032

[31] Ceri H, Olson ME, Stremick C, Read RR, Morck D, Buret A. 1999. The calgary biofilm device: new technology for rapid determination of antibiotic susceptibilities of bacterial biofilms. J Clin Microbiol 37:1771–1776. doi:10.1128/JCM.37.6.1771-1776.1999 10325322 PMC84946

[32] Girard LP, Ceri H, Gibb AP, Olson M, Sepandj F. 2010. MIC versus MBEC to determine the antibiotic sensitivity of Staphylococcus aureus in peritoneal dialysis peritonitis. Perit Dial Int 30:652–656. doi:10.3747/pdi.2010.00010 21148059

[33] Donlan RM, Costerton JW. 2002. Biofilms: survival mechanisms of clinically relevant microorganisms. Clin Microbiol Rev 15:167–193. doi:10.1128/CMR.15.2.167-193.2002 11932229 PMC118068

[34] Leid JG, Shirtliff ME, Costerton JW, Stoodley P. 2002. Human leukocytes adhere to, penetrate, and respond to Staphylococcus aureus biofilms. Infect Immun 70:6339–6345. doi:10.1128/IAI.70.11.6339-6345.2002 12379713 PMC130380

[35] Schommer NN, Christner M, Hentschke M, Ruckdeschel K, Aepfelbacher M, Rohde H. 2011. Staphylococcus epidermidis uses distinct mechanisms of biofilm formation to interfere with phagocytosis and activation of mouse macrophage-like cells 774A.1. Infect Immun 79:2267–2276. doi:10.1128/IAI.01142-10 21402760 PMC3125858

[36] Scherr TD, Hanke ML, Huang O, James DBA, Horswill AR, Bayles KW, Fey PD, Torres VJ, Kielian T. 2015. Staphylococcus aureus biofilms induce macrophage dysfunction through leukocidin AB and alpha-toxin. mBio 6:e01021-15. doi:10.1128/mBio.01021-15 26307164 PMC4550693

[37] Crosby HA, Kwiecinski J, Horswill AR. 2016. Staphylococcus aureus aggregation and coagulation mechanisms, and their function in host–pathogen interactions. Adv Appl Microbiol 96:1–41. doi:10.1016/bs.aambs.2016.07.018 27565579 PMC5221605

[38] Geoghegan JA, Corrigan RM, Gruszka DT, Speziale P, O’Gara JP, Potts JR, Foster TJ. 2010. Role of surface protein SasG in biofilm formation by Staphylococcus aureus. J Bacteriol 192:5663–5673. doi:10.1128/JB.00628-10 20817770 PMC2953683

[39] Corrigan RM, Rigby D, Handley P, Foster TJ. 2007. The role of Staphylococcus aureus surface protein SasG in adherence and biofilm formation. Microbiology (Reading) 153:2435–2446. doi:10.1099/mic.0.2007/006676-0 17660408

[40] Conrady DG, Brescia CC, Horii K, Weiss AA, Hassett DJ, Herr AB. 2008. A zinc-dependent adhesion module is responsible for intercellular adhesion in staphylococcal biofilms. Proc Natl Acad Sci U S A 105:19456–19461. doi:10.1073/pnas.0807717105 19047636 PMC2592360

[41] Macintosh RL, Brittan JL, Bhattacharya R, Jenkinson HF, Derrick J, Upton M, Handley PS. 2009. The terminal A domain of the fibrillar accumulation-associated protein (Aap) of Staphylococcus epidermidis mediates adhesion to human corneocytes. J Bacteriol 191:7007–7016. doi:10.1128/JB.00764-09 19749046 PMC2772481

[42] Roche FM, Meehan M, Foster TJ. 2003. The Staphylococcus aureus surface protein SasG and its homologues promote bacterial adherence to human desquamated nasal epithelial cells. Microbiology (Reading) 149:2759–2767. doi:10.1099/mic.0.26412-0 14523109

[43] Rohde H, Burdelski C, Bartscht K, Hussain M, Buck F, Horstkotte MA, Knobloch J-M, Heilmann C, Herrmann M, Mack D. 2005. Induction of Staphylococcus epidermidis biofilm formation via proteolytic processing of the accumulation-associated protein by staphylococcal and host proteases. Mol Microbiol 55:1883–1895. doi:10.1111/j.1365-2958.2005.04515.x 15752207

[44] Speziale P, Pietrocola G, Foster TJ, Geoghegan JA. 2014. Protein-based biofilm matrices in staphylococci. Front Cell Infect Microbiol 4:171. doi:10.3389/fcimb.2014.00171 25540773 PMC4261907

[45] Conrady DG, Wilson JJ, Herr AB. 2013. Structural basis for Zn2+-dependent intercellular adhesion in staphylococcal biofilms. Proc Natl Acad Sci U S A 110:E202–E211. doi:10.1073/pnas.1208134110 23277549 PMC3549106

[46] Paharik AE, Kotasinska M, Both A, Hoang T-MN, Büttner H, Roy P, Fey PD, Horswill AR, Rohde H. 2017. The metalloprotease SepA governs processing of accumulation-associated protein and shapes intercellular adhesive surface properties in Staphylococcus epidermidis. Mol Microbiol 103:860–874. doi:10.1111/mmi.13594 27997732 PMC5480372

[47] Meyer TC, Michalik S, Holtfreter S, Weiss S, Friedrich N, Völzke H, Kocher T, Kohler C, Schmidt F, Bröker BM, Völker U. 2021. A comprehensive view on the human antibody repertoire against Staphylococcus aureus antigens in the general population. Front Immunol 12:651619. doi:10.3389/fimmu.2021.651619 33777051 PMC7987813

[48] Crosby HA, Schlievert PM, Merriman JA, King JM, Salgado-Pabón W, Horswill AR. 2016. The Staphylococcus aureus global regulator MgrA modulates clumping and virulence by controlling surface protein expression. PLOS Pathog 12:e1005604. doi:10.1371/journal.ppat.1005604 27144398 PMC4856396

[49] Mills KB, Maciag JJ, Wang C, Crawford JA, Enroth TJ, Keim KC, Dufrêne YF, Robinson DA, Fey PD, Herr AB, Horswill AR. 2023. Staphylococcus aureus skin colonization is mediated by SasG lectin variation. bioRxiv:2023.11.20.567970. doi:10.1101/2023.11.20.567970 PMC11866565 38568806

[50] Monecke S, Coombs G, Shore AC, Coleman DC, Akpaka P, Borg M, Chow H, Ip M, Jatzwauk L, Jonas D, Kadlec K, Kearns A, Laurent F, O’Brien FG, Pearson J, Ruppelt A, Schwarz S, Scicluna E, Slickers P, Tan H-L, Weber S, Ehricht R. 2011. A field guide to pandemic, epidemic and sporadic clones of methicillin-resistant Staphylococcus aureus. PLoS One 6:e17936. doi:10.1371/journal.pone.0017936 21494333 PMC3071808

[51] Crosby HA, Tiwari N, Kwiecinski JM, Xu Z, Dykstra A, Jenul C, Fuentes EJ, Horswill AR. 2020. The Staphylococcus aureus ArlRS two-component system regulates virulence factor expression through MgrA. Mol Microbiol 113:103–122. doi:10.1111/mmi.14404 31618469 PMC7175635

[52] Kollef MH. 1999. The prevention of ventilator-associated pneumonia. N Engl J Med 340:627–634. doi:10.1056/NEJM199902253400807 10029648

[53] Scannapieco FA. 1999. Role of oral bacteria in respiratory infection. J Periodontol 70:793–802. doi:10.1902/jop.1999.70.7.793 10440642

[54] Scannapieco FA, Wang B, Shiau HJ. 2001. Oral bacteria and respiratory infection: effects on respiratory pathogen adhesion and epithelial cell proinflammatory cytokine production. Ann Periodontol 6:78–86. doi:10.1902/annals.2001.6.1.78 11887474

[55] Dong J, Li W, Wang Q, Chen J, Zu Y, Zhou X, Guo Q. 2021. Relationships between oral microecosystem and respiratory diseases. Front Mol Biosci 8:718222. doi:10.3389/fmolb.2021.718222 35071321 PMC8767498

[56] Wörmann ME, Reichmann NT, Malone CL, Horswill AR, Gründling A. 2011. Proteolytic cleavage inactivates the Staphylococcus aureus lipoteichoic acid synthase. J Bacteriol 193:5279–5291. doi:10.1128/JB.00369-11 21784926 PMC3187375

[57] Mills KB, Jia F, Stein ME, Keim KC, Davidson RM, Horswill AR. 2022. Genome sequences of two methicillin-sensitive Staphylococcus aureus healthy skin isolates. Microbiol Resour Announc 11:e0040222. doi:10.1128/mra.00402-22 35575560 PMC9202384

[58] Blanchard AA, Ezzati P, Shamshurin D, Nistor AC, Leygue E, Wilkins JA, Myal Y. 2015. Towards further defining the proteome of mouse saliva. Proteome Sci 13:10. doi:10.1186/s12953-015-0068-3 25762866 PMC4355469

[59] Karn RC, Chung AG, Laukaitis CM. 2013. Shared and unique proteins in human, mouse and rat saliva proteomes: footprints of functional adaptation. Proteomes 1:275–289. doi:10.3390/proteomes1030275 24926433 PMC4051352

[60] Sales KU, Hobson JP, Wagenaar-Miller R, Szabo R, Rasmussen AL, Bey A, Shah MF, Molinolo AA, Bugge TH. 2011. Expression and genetic loss of function analysis of the HAT/DESC cluster proteases TMPRSS11A and HAT. PLoS One 6:e23261. doi:10.1371/journal.pone.0023261 21853097 PMC3154331

[61] Szabo R, Bugge TH. 2020. Membrane-anchored serine proteases as regulators of epithelial function. Biochem Soc Trans 48:517–528. doi:10.1042/BST20190675 32196551 PMC9869603

[62] Nilsson P, Ripa T. 2006. Staphylococcus aureus throat colonization is more frequent than colonization in the anterior nares. J Clin Microbiol 44:3334–3339. doi:10.1128/JCM.00880-06 16954269 PMC1594670

[63] Gustafsson EB, Ringberg H, Johansson PJH. 2007. MRSA in children from foreign countries adopted to Swedish families. Acta Paediatr 96:105–108. doi:10.1111/j.1651-2227.2007.00096.x 17187614

[64] Hamdan-Partida A, Sainz-Espuñes T, Bustos-Martínez J. 2010. Characterization and persistence of Staphylococcus aureus strains isolated from the anterior nares and throats of healthy carriers in a Mexican community. J Clin Microbiol 48:1701–1705. doi:10.1128/JCM.01929-09 20335416 PMC2863913

[65] Paling FP, Hazard D, Bonten MJM, Goossens H, Jafri HS, Malhotra-Kumar S, Sifakis F, Weber S, Kluytmans JAJW, ASPIRE-ICU Study Team. 2020. Association of Staphylococcus aureus colonization and pneumonia in the intensive care unit. JAMA Netw Open 3:e2012741. doi:10.1001/jamanetworkopen.2020.12741 32997125 PMC7527877

[66] Paling FP, Wolkewitz M, Bode LGM, Klein Klouwenberg PMC, Ong DSY, Depuydt P, de Bus L, Sifakis F, Bonten MJM, Kluytmans J. 2017. Staphylococcus aureus colonization at ICU admission as a risk factor for developing S. aureus ICU pneumonia. Clin Microbiol Infect 23:49. doi:10.1016/j.cmi.2016.09.022 27693658

[67] Kukita K, Kawada-Matsuo M, Oho T, Nagatomo M, Oogai Y, Hashimoto M, Suda Y, Tanaka T, Komatsuzawa H. 2013. Staphylococcus aureus SasA is responsible for binding to the salivary agglutinin gp340, derived from human saliva. Infect Immun 81:1870–1879. doi:10.1128/IAI.00011-13 23439307 PMC3676022

[68] Heo S-M, Choi K-S, Kazim LA, Reddy MS, Haase EM, Scannapieco FA, Ruhl S. 2013. Host defense proteins derived from human saliva bind to Staphylococcus aureus. Infect Immun 81:1364–1373. doi:10.1128/IAI.00825-12 23403559 PMC3639616

[69] Foster TJ, Geoghegan JA, Ganesh VK, Höök M. 2014. Adhesion, invasion and evasion: the many functions of the surface proteins of Staphylococcus aureus. Nat Rev Microbiol 12:49–62. doi:10.1038/nrmicro3161 24336184 PMC5708296

[70] Toledo-Arana A, Merino N, Vergara-Irigaray M, Débarbouillé M, Penadés JR, Lasa I. 2005. Staphylococcus aureus develops an alternative, ica-independent biofilm in the absence of the arlRS two-component system. J Bacteriol 187:5318–5329. doi:10.1128/JB.187.15.5318-5329.2005 16030226 PMC1196035

[71] Kwiecinski JM, Crosby HA, Valotteau C, Hippensteel JA, Nayak MK, Chauhan AK, Schmidt EP, Dufrêne YF, Horswill AR. 2019. Staphylococcus aureus adhesion in endovascular infections is controlled by the ArlRS–MgrA signaling cascade. PLOS Pathog 15:e1007800. doi:10.1371/journal.ppat.1007800 31116795 PMC6548404

[72] Jackson MS. 2000. Staphylococci in the oral flora of healthy children and those receiving treatment for malignant disease. Microb Ecol Health Dis 12:60–64. doi:10.1080/089106000435617

[73] Heo SM, Ruhl S, Scannapieco FA. 2013. Implications of salivary protein binding to commensal and pathogenic bacteria. J Oral Biosci 55:169–174. doi:10.1016/j.job.2013.06.004 24707190 PMC3974197

[74] Fingleton B, Menon R, Carter KJ, Overstreet PD, Hachey DL, Matrisian LM, McIntyre JO. 2004. Proteinase activity in human and murine saliva as a biomarker for proteinase inhibitor efficacy. Clin Cancer Res 10:7865–7874. doi:10.1158/1078-0432.CCR-04-1252 15585619

[75] Niederman MS, Cilloniz C. 2022. Aspiration pneumonia. Rev Esp Quimioter 35:73–77. doi:10.37201/req/s01.17.2022 35488832 PMC9106188

[76] Yonemoto K, Chiba A, Sugimoto S, Sato C, Saito M, Kinjo Y, Marumo K, Mizunoe Y. 2019. Redundant and distinct roles of secreted protein Eap and cell wall-anchored protein SasG in biofilm formation and pathogenicity of Staphylococcus aureus. Infect Immun 87:e00894-18. doi:10.1128/IAI.00894-18 30670553 PMC6434138

[77] Monk IR, Shah IM, Xu M, Tan M-W, Foster TJ. 2012. Transforming the untransformable: application of direct transformation to manipulate genetically Staphylococcus aureus and Staphylococcus epidermidis. mBio 3:e00277-11. doi:10.1128/mBio.00277-11 22434850 PMC3312211

[78] Nair D, Memmi G, Hernandez D, Bard J, Beaume M, Gill S, Francois P, Cheung AL. 2011. Whole-genome sequencing of Staphylococcus aureus strain RN4220, a key laboratory strain used in virulence research, identifies mutations that affect not only virulence factors but also the fitness of the strain. J Bacteriol 193:2332–2335. doi:10.1128/JB.00027-11 21378186 PMC3133102

[79] Baba T, Takeuchi F, Kuroda M, Yuzawa H, Aoki K, Oguchi A, Nagai Y, Iwama N, Asano K, Naimi T, Kuroda H, Cui L, Yamamoto K, Hiramatsu K. 2002. Genome and virulence determinants of high virulence community-acquired MRSA. Lancet 359:1819–1827. doi:10.1016/s0140-6736(02)08713-5 12044378

[80] Boles BR, Thoendel M, Roth AJ, Horswill AR. 2010. Identification of genes involved in polysaccharide-independent Staphylococcus aureus biofilm formation. PLoS One 5:e10146. doi:10.1371/journal.pone.0010146 20418950 PMC2854687

[81] Baba T, Bae T, Schneewind O, Takeuchi F, Hiramatsu K. 2008. Genome sequence of Staphylococcus aureus strain Newman and comparative analysis of staphylococcal genomes: polymorphism and evolution of two major pathogenicity islands. J Bacteriol 190:300–310. doi:10.1128/JB.01000-07 17951380 PMC2223734

[82] Kuroda M, Ohta T, Uchiyama I, Baba T, Yuzawa H, Kobayashi I, Cui L, Oguchi A, Aoki K, Nagai Y, et al.. 2001. Whole genome sequencing of meticillin-resistant Staphylococcus aureus. Lancet 357:1225–1240. doi:10.1016/s0140-6736(00)04403-2 11418146

[83] Schlievert PM, Blomster DA. 1983. Production of staphylococcal pyrogenic exotoxin type C: influence of physical and chemical factors. J Infect Dis 147:236–242. doi:10.1093/infdis/147.2.236 6827140

[84] Holden MTG, Feil EJ, Lindsay JA, Peacock SJ, Day NPJ, Enright MC, Foster TJ, Moore CE, Hurst L, Atkin R, et al.. 2004. Complete genomes of two clinical Staphylococcus aureus strains: evidence for the rapid evolution of virulence and drug resistance. Proc Natl Acad Sci U S A 101:9786–9791. doi:10.1073/pnas.0402521101 15213324 PMC470752

[85] Bateman BT, Donegan NP, Jarry TM, Palma M, Cheung AL. 2001. Evaluation of a tetracycline-inducible promoter in Staphylococcus aureus in vitro and in vivo and its application in demonstrating the role of sigB in microcolony formation. Infect Immun 69:7851–7857. doi:10.1128/IAI.69.12.7851-7857.2001 11705967 PMC98881

[86] Corrigan RM, Foster TJ. 2009. An improved tetracycline-inducible expression vector for Staphylococcus aureus. Plasmid 61:126–129. doi:10.1016/j.plasmid.2008.10.001 18996145

[87] Pang YY, Schwartz J, Thoendel M, Ackermann LW, Horswill AR, Nauseef WM. 2010. agr-dependent interactions of Staphylococcus aureus USA300 with human polymorphonuclear neutrophils. J Innate Immun 2:546–559. doi:10.1159/000319855 20829608 PMC2982852

[88] Rocco CJ, Dennison KL, Klenchin VA, Rayment I, Escalante-Semerena JC. 2008. Construction and use of new cloning vectors for the rapid isolation of recombinant proteins from Escherichia coli. Plasmid 59:231–237. doi:10.1016/j.plasmid.2008.01.001 18295882 PMC2386272

[89] Löfblom J, Kronqvist N, Uhlén M, Ståhl S, Wernérus H. 2007. Optimization of electroporation-mediated transformation: Staphylococcus carnosus as model organism. J Appl Microbiol 102:736–747. doi:10.1111/j.1365-2672.2006.03127.x 17309623

[90] Novick RP. 1991. Genetic systems in staphylococci. Methods Enzymol 204:587–636. doi:10.1016/0076-6879(91)04029-n 1658572

[91] Bankevich A, Nurk S, Antipov D, Gurevich AA, Dvorkin M, Kulikov AS, Lesin VM, Nikolenko SI, Pham S, Prjibelski AD, Pyshkin AV, Sirotkin AV, Vyahhi N, Tesler G, Alekseyev MA, Pevzner PA. 2012. SPAdes: a new genome assembly algorithm and its applications to single-cell sequencing. J Comput Biol 19:455–477. doi:10.1089/cmb.2012.0021 22506599 PMC3342519

[92] Gurevich A, Saveliev V, Vyahhi N, Tesler G. 2013. QUAST: quality assessment tool for genome assemblies. Bioinformatics 29:1072–1075. doi:10.1093/bioinformatics/btt086 23422339 PMC3624806

[93] Seemann T. 2014. Prokka: rapid prokaryotic genome annotation. Bioinformatics 30:2068–2069. doi:10.1093/bioinformatics/btu153 24642063

[94] Scotto-Lavino E, Du G, Frohman MA. 2006. 5' end cDNA amplification using classic RACE. Nat Protoc 1:2555–2562. doi:10.1038/nprot.2006.480 17406509

[95] Luong TT, Lee CY. 2007. Improved single-copy integration vectors for Staphylococcus aureus. J Microbiol Methods 70:186–190. doi:10.1016/j.mimet.2007.04.007 17512993 PMC2001203

