# Supplementary material for: Host-derived protease promotes aggregation of Staphylococcus aureus by cleaving the surface protein SasG
Source: mBio. 2024 Mar 21;15(4):e03483-23. doi: 10.1128/mbio.03483-23 (PMC11005337; doi:10.1128/mbio.03483-23)
Supplement: Supplemental Figures — Figures S1-S3. [file mbio.03483-23-s0001.docx]

**Supplementary Figures**

**
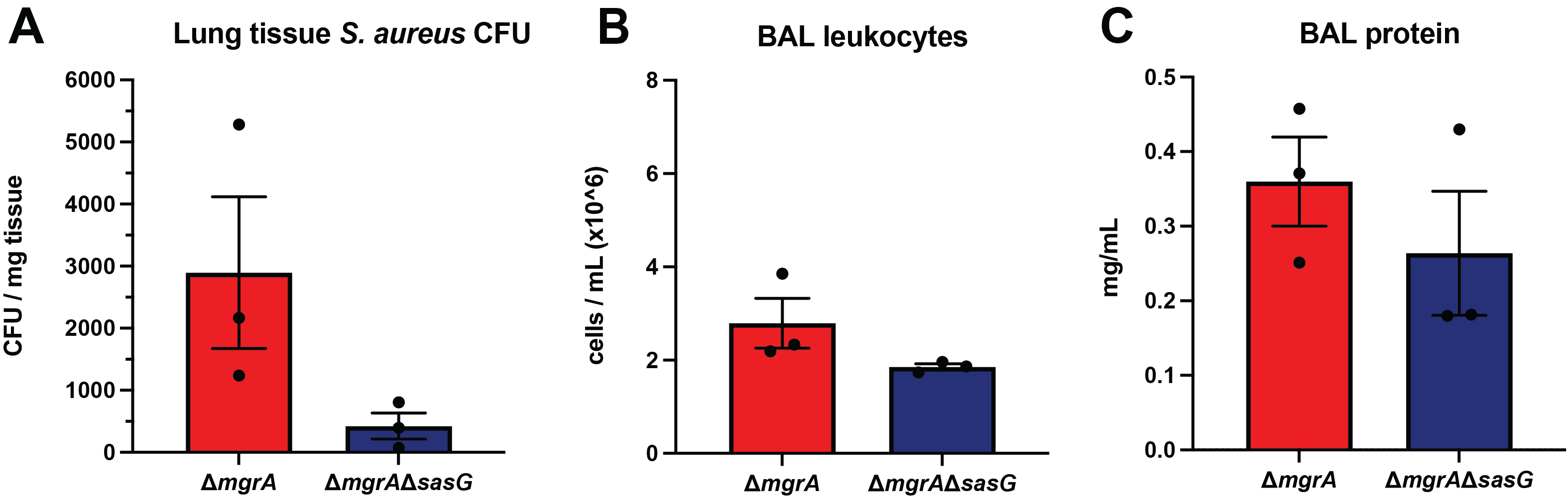
**

# Supplemental Fig. 1

Mice were infected intratracheally as shown in Fig. 6, but with a lower dose of *S. aureus* MW2 *ΔmgrA* and its congenic strain *ΔmgrA ΔsasG* lacking SasG. Pneumonia severity outcomes were assessed 24hrs following infection. The same trends were observed at the lower dose as those in Fig. 6. Compared to the *ΔmgrA* strain expressing SasG, mice infected with the double mutant lacking SasG had decreased CFU recovered from lung homogenates (A) and no significant differences in leukocyte recruitment (B) or protein levels in lavage fluid (C). Results presented as means ± SEM.

**
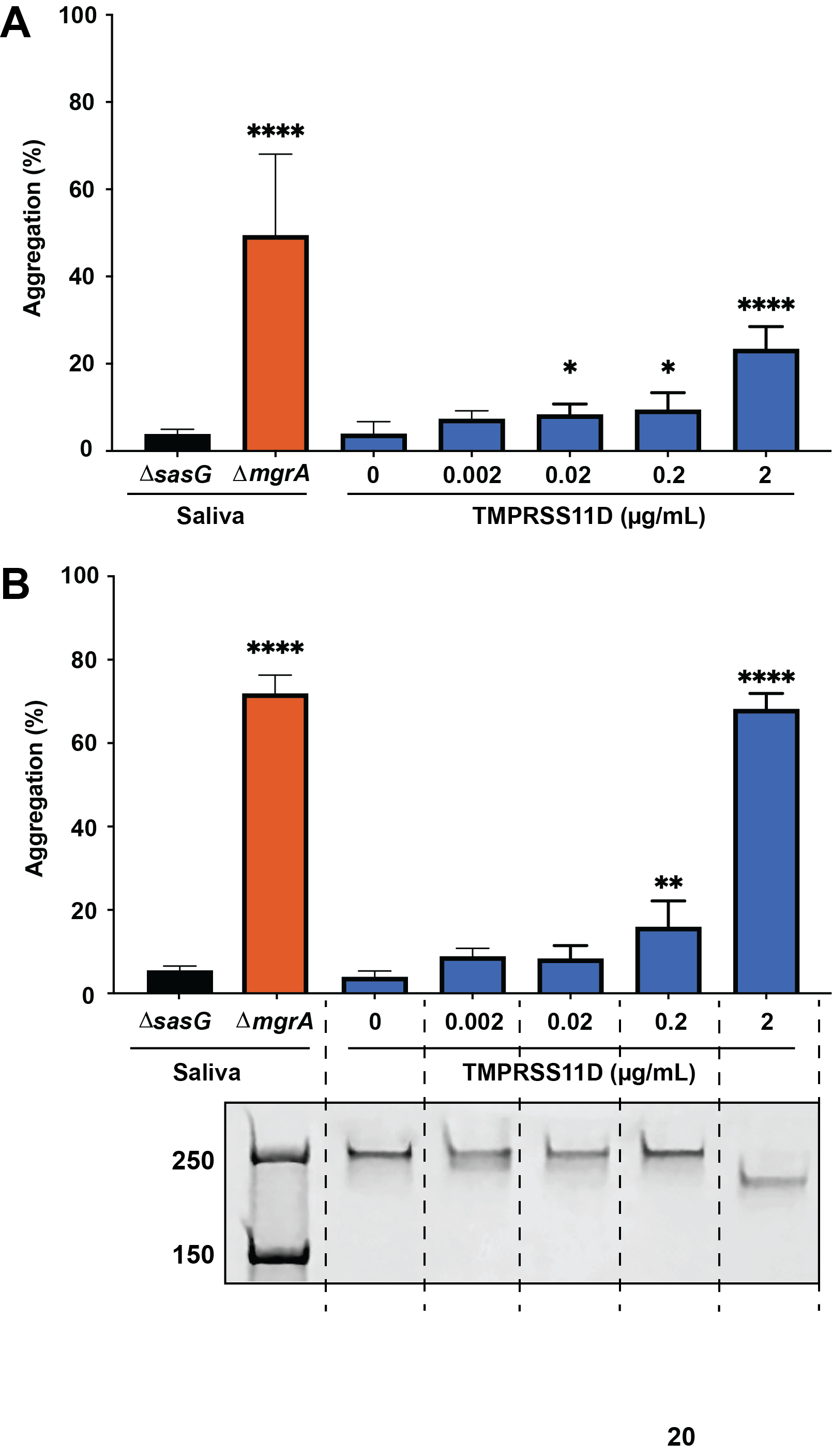
**

**Supplementary Fig. 2**.

Murine airway trypsin-like protease (TMPRSS11D) can process SasG and promote *S. aureus* aggregation. *S. aureus* (A) 502a or (B) MW2 *mgrA* and *sasG* mutant strains were resuspended in either saliva or PBS supplemented with recombinant TMPRSS11D and allowed to aggregate for 1 h. (C) Purified full-length SasG was incubated for 1 h with either human saliva or serial dilutions of TMPRSS11D before running on an SDS-PAGE gel and staining with Coomassie. Measurements are averages and standard deviations of three separate experiments Significance was calculated by One-Way ANOVA ****p<0.0001.

**
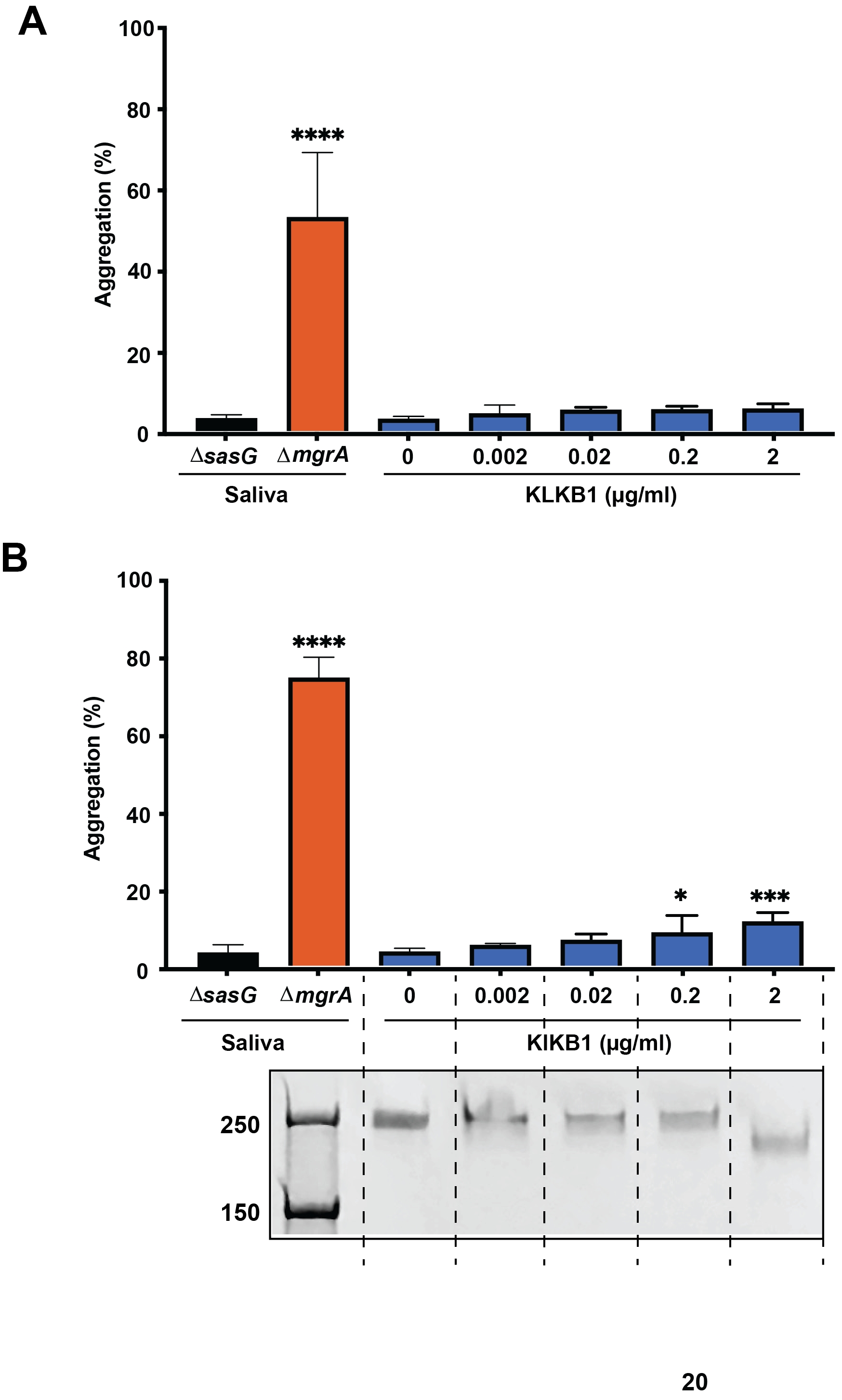
**

**;**

**Supplementary Fig. 3**. Murine Kallikrein 1 (KLKB1) can process SasG and promotes limited *S. aureus* aggregation. *S. aureus* (A) 502a or (B) MW2 *mgrA* and *sasG* mutant strains were resuspended in either saliva or PBS supplemented with recombinant KLKB1 and allowed to aggregate for 1 h. (C) Purified full-length SasG was incubated for 1 h with either human saliva or serial dilutions of KLKB1 before running on an SDS-PAGE gel and staining with Coomassie. Measurements are averages and standard deviations of three separate experiments Significance was calculated by One-Way ANOVA ****p<0.0001
