# Supplementary material for: Host-derived protease promotes aggregation of Staphylococcus aureus by cleaving the surface protein SasG
Source: mBio. 2024 Mar 21;15(4):e03483-23. doi: 10.1128/mbio.03483-23 (PMC11005337; doi:10.1128/mbio.03483-23)
Supplement: Supplemental Table — Table S1. [file mbio.03483-23-s0002.docx]

**Supplemental Table 1**

| **Protease** | **sequence coverage (%)** | **MW** | **pI** | **Type** |
| --- | --- | --- | --- | --- |
| Trypsin-1 | 15 | 24.1 | 7.64 | serine |
| Prostasin | 6 | 33.3 | 5.40 | trypsin-type serine |
| Serine protease 27 | 7 | 28.2 | 6.07 | serine |
| Cathepsin D | 40 | 37.9 | 5.60 | aspartic |
| Cathepsin B | 22 | 27.8 | 5.22 | cysteine |
| Cathepsin H | 7 | 24.2 | 5.93 | cysteine |
| Cathepsin Z | 18 | 27.1 | 5.48 | cysteine |
